# Cordycepin from *Cordyceps militaris* ameliorates diabetic nephropathy via the miR-193b-5p/MCL-1 axis

**DOI:** 10.1186/s13020-023-00842-5

**Published:** 2023-10-13

**Authors:** Rong Zheng, Weijie Zhang, Jufang Song, Yifei Zhong, Rong Zhu

**Affiliations:** Department of Nephrology, Longhua Hospital, Shanghai University of Traditional Chinese Medicine, 725 South Wanping Road, Shanghai, 200032 China

**Keywords:** Cordycepin (CRD), Diabetic nephropathy (DN), miR-193b-5p/MCL-1 axis, Inflammation, Oxidative stress

## Abstract

**Background:**

Diabetic nephropathy (DN) is a chronic kidney disease that develops in patients with diabetes mellitus. Cordycepin (CRD), a secondary metabolite produced by *Cordyceps militaris*, has a variety of bioactive properties. In this study, DN mice and high glucose (HG)-treated HK-2 were used to evaluate the diagnostic value of CRD.

**Methods:**

Quantitative real-time PCR (qRT-PCR), western blotting, immunofluorescence analysis, and immunohistochemical staining were used to assess changes in mRNA and protein expression. Oxidative stress was evaluated by detecting the production of reactive oxygen species (ROS) and the activity of antioxidant enzymes. Cell apoptosis was detected by the TUNEL and flow cytometric methods. The interaction of miR-193b-5p and myeloid leukemia 1 (MCL-1) was examined by bioinformatics analysis and luciferase reporter assay. The protective effects of CRD on DN mice were evaluated by examining DN related biochemical indicators and renal histopathology.

**Results:**

In response to HG, the level of miR-193b-5p was elevated, whilst the level of MCL-1 was downregulated, and CRD therapy reversed this behavior. MCL-1 was further identified to be miR-193b-5p target. CRD attenuated HG-induced cell damage, inflammation and abnormal energy metabolism. Mechanistic investigations on in vitro models confirmed that protective effect of CRD against HG challenge to HK-2 cells is mediated through the regulation of expression of miR-193b-5p/MCL-1 axis. By examining DN related biochemical markers and renal histopathology, the protective effects of CRD on DN mice was assessed.

**Conclusions:**

In summary, CRD decreased oxidative stress and inflammation by increasing miR-193b-5p and inactivating downstream MCL-1 in DN, hinting the pivotal values of CRD and miR-193b-5p in the management of DN.

**Supplementary Information:**

The online version contains supplementary material available at 10.1186/s13020-023-00842-5.

## Introduction

Diabetes Mellitus (DM), one of the world’s leading chronic diseases, is caused by partial insulin resistance or full insulin insufficiency. Due to interactions among multiple complex factors such as genetic, environment and lifestyle factors, the disease has become recently more prevalent. Diabetes is further amplified by inflammation, atherosclerosis and dyslipidemia. Patients must maintain their glycemic levels through food and treatment in order to avoid adverse effects [1–3]. If glycemic levels are not maintained, many secondary consequences such as cardiovascular diseases, retinopathy, nephropathy, and neuropathy occurs [3, 4]. When considering treatment options, many alternatives from oral to injectables are currently available in the market [5]. Treatment options targeting secondary consequences would not only allow maintenance of the disease in patients with severe states of the disease but also allow elucidating disease biology.

Diabetic nephropathy (DN) is one of the most prevalent and severe secondary effects, resulting in increased mortality and morbidity among diabetic patients [6]. With the prevalence of diabetes rising, the need for therapy and care is crucial because one-third of diabetic patients experience DN [7]. Diagnosis of DN is complicated by several factors. To begin with, most individuals do not develop the classic form of DN. Frequently observed clinical manifestations encompass proteinuria, elevated blood pressure, and diminished glomerular filtration. However, it is noteworthy that non-proteinuria is a prevalent occurrence among individuals diagnosed with DN [7, 8]. Similarly, renin-angiotensin blockade therapy is considered only after the onset of albuminuria. However, many patients do not develop albuminuria, hence the timing for initiating therapeutic measures has become increasingly controversial [9]. Due to the factors described above, it is clear that there is a need for novel diagnostic markers and therapeutic strategies for DN.

Since its anti-tumor, anti-inflammatory, and antiviral properties were found, Cordycepin (3′-deoxyadenosine, CRD), a bioactive molecule found in *Cordyceps militaris* (*C. militaris*), has attracted a lot of interest [10]. In mammalian cells, CRD is involved in a number of different cellular processes, including signal transductions [11], apoptosis [12], the cell cycle [13], and the formation of reactive oxygen species (ROS) [14]. A recent study found that the physiological function of CRD has some therapeutic benefits for treating DN in rats via inducing autophagy [15].

MicroRNAs (miRNAs) are short non-coding RNA (20 nt) which bind to the 3′ UTR of a target mRNA and either activate or deactivate the corresponding mRNA [16]. Initially, thought to be non-functional, recent studies have highlighted their role in many biological processes including cell proliferation, differentiation, maturation, apoptosis and metabolism [17]. Many miRNAs have been associated with microvascular complications of DM such as nephropathy, neuropathy and retinopathy [18]. In DN, many miRNAs such as miR-126 [19], miR-29b [20], and miR-337 [21] have been identified to be key players in binding and regulating downstream targets. Interestingly, miRNAs have also been considered as a key biomarker in diagnosing DN [22].

In this work, we discovered that miR-193b-5p, one of these miRNAs, was significantly disrupted in DN. Its dysregulation was proven in DN samples taken from both in vivo and in vitro experiments. We also made an effort to look into the effects of its deregulation at the downstream level and discovered that miR-193b-5p binds to and regulates the expression of its downstream target, myeloid leukemia 1 (MCL-1), which modifies the development of DN. Additionally, it was discovered that CRD, dramatically upregulates miR-193b-5p and shields HG-exposed HK-2 cells from inflammatory response, oxidative stress, and apoptosis. As a result, our study aids in the discovery of new DN biomarkers and targets and sheds light on the potential application of CRD as a therapeutic substitute for DN.

## Materials and methods

### Patients

Patients diagnosed with DN (n = 50) and healthy volunteers (n = 50) from the general population willing to undergo physical examinations were enrolled in this study at the Longhua Hospital during the period extending from December 2020 to March 2022. Patients diagnosed with DN were separated into two groups: those with microalbuminuria (UAER between 30 mg/24 h and 300 mg/24 h) and those with macroalbuminuria (UAER over 300 mg/24 h). Table 1 displays the clinical characteristics of the DN participants that were studied. Blood was collected from patients as well as normal healthy volunteers and centrifuged to obtain serum samples, which were then kept in a refrigerator at − 80 °C until further experimental procedures. Written informed consent was obtained from each of the participant. Our research was approved by the Ethics Committee of Longhua Hospital, Shanghai University of Traditional Chinese Medicine.

**Table 1:** Characteristics of the participants stratified

Clinical parameter	Normal N = 50	T2DM N = 27	T2DM-DN N = 23	F	Q value
Age	51.22 ± 7.14	63.18 ± 8.90	65.91 ± 10.30	0.65	0.5211
Sex (male, %)	62	59.2	65.2	–	–
BMI (kg/m^2^)	24.22 ± 2.02	25.32 ± 3.96	25.69 ± 3.08	2.49	0.0876
FBG (mg/dL)	88.53 ± 9.65	158.32 ± 17.45^a^**	163.68 ± 16.89^b^**	338.43	0.000**
TG (mg/dL)	105.23 ± 29.63	162.38 ± 45.25^a^**	164.32 ± 34.23^b^**	33.49	0.0000**
TC (mg/dL)	113.26 ± 24.53	165.84 ± 41.02^a^**	176.27 ± 37.7^b^**	38.81	0.000**
Urea (mg/dL)	19.36 ± 5.36	28.45 ± 12.59^a^*	96.17 ± 35.28^b^**^, c^**	143.59	0.000**
Creatinine (mg/dL)	0.96 ± 0.24	0.85 ± 0.15^a^**	2.36 ± 1.36^b^**^, c^**	40.54	0.000**
eGFR (mL/min per 1.73 m^2^)	103.38 ± 27.89	89.63 ± 24.17^a^*	46.32 ± 15.32^b^**^, c^**	42.84	0.000**
DPB (mmHg)	84.2 ± 9.3	87.6 ± 10.1	86.3 ± 9.6	1.18	0.31
PP (mmHg)	51.3 ± 6.3	54.6 ± 7.9	53.5 ± 7.7	2.09	0.13
AST (U/L)	17.55 ± 5.05	20.98 ± 6.14^a^*	21.23 ± 7.05^b^*	4.6351	0.012*
ALT (U/L)	18.03 ± 4.48	21.33 ± 5.05^a^*	23.08 ± 6.96^b^**	8.21	0.0005**
Cr (U/L)	1.02 ± 0.13	1.29 ± 0.15^a^**	2.23 ± 0.63b^**, c^**	111.43	0.000**
LDL-C (mg/dL)	109.84 ± 11.63	103.68 ± 14.87	110.68 ± 13.82	2.41	0.095
HDL-C (mg/dL)	53.24 ± 7.59	55.36 ± 9.87	56.32 ± 9.87	1.20	0.306

*BMI* body mass index, *FBG* fasting blood glucose, *TG* triglyceride, *TC* total-cholesterol, *eGFR* estimated glomerular filtration rate, *DPB* diastolic blood pressure, *SBP* systolic blood pressure, *PP* pulse pressure, *AST* aspartate aminotransferase, *ALT* alanine aminotransferase, *Cr* creatinine, *LDL-C* low density lipoprotein-cholesterol, *HDL-C* high density lipoprotein-cholesterol

*P < 0.05, **P < 0.01

^a^Comparison between Normal and T2DM

^b^Comparison between normal and T2DM-DN

^c^Comparison between T2DM and T2DM-DN

### Animal experiment

All procedures using animals in this study were performed according to the protocol approved by the Institutional Animal Care and Use Committee of Longhua Hospital, Shanghai University of Traditional Chinese Medicine, China. The animal ethics number is 20180702A. Eight-week-old male C57BLKS/J db/db and C57BLKS/J db/m mice obtained from the Aier Matt Experimental Animal Company (Suzhou, China). The mice were adapted for 2 weeks in specific situations. The db/m mice were randomly divided into two groups (n = 8 per group): normal group (db/m group) and normal + CRD group (400 mg/kg) (db/m + CRD group), while db/db mice were assigned into five groups (n = 8 per group): (1) DN group, (2) DN + glibenclamide group (Gli, 5 mg/kg, DN + Gli group), (3) DN + CRD group (400 mg/kg), (4) DN + CRD (400 mg/kg) + inhibitor NC group, and (5) DN + CRD (400 mg/kg) + miR-193b-5p inhibitor group. The mice in the CRD treatment groups were administrated by means of intragastric administration with indicated dose of CRD. The mice in db/m group were given the same dose of normal saline for gavage. The CRD treatment lasted for the next 12 weeks. MiR-193b-5p inhibitor or its negative control inhibitor NC was intravenously injected into the tail vein of db/db mice at 9th week intravenously via tail vein at doses of 5 µg/mice three times per week for 4 weeks. During the study, fasting blood glucose (FBG) was measured weekly, the 24 h urine from the mice was collected in metabolic cages to quantify urinary protein. All mice were fasted for 12 h to sacrifice under anesthesia, blood samples and renal tissues were collected to measure corresponding biochemical parameters and renal pathological changes, the kidney weight (KW) was also recorded. Serum was isolated from whole blood samples through centrifugation (3000 rpm for 10 min). Serum creatinine (Scr) or blood urea nitrogen (BUN) level was respectively detected using Creatinine Colorimetric Assay Kit (Cayman Chemical) or BUN detection kit (StressMarq Biosciences, Victoria, British Columbia) following the instructions of the manufacturer.

### Immunofluorescence

The cells (3 × 10^4^) were cultured on coverslips in 35 mm dishes. The preparations were washed with phosphate-buffered saline (PBS) and fixed with 3.7% formaldehyde for 30 min. The cells were washed with PBS again and permeabilized with 0.5% Triton X-100 for 10 min. After this time, the cells were washed with PBS, and the primary antibodies (MCL-1, Abcam, ab32087, 1:500) were added. The incubations with antibodies were performed in a humidified chamber overnight. The cells were washed with PBS again, and the secondary antibody was added and incubated for 3 h. The images were obtained using a confocal laser-scanning microscope (LSM 510-ZEISS).

### Immunohistochemistry

Kidney tissues were initially fixed in 4% paraformaldehyde and further embedded in paraffin. Tissues were sectioned at 5 μm thickness, deparaffinized with xylene and rehydrated with alcohol in a gradient manner. Internal peroxidase activity was inactivated using 3% hydrogen peroxide in 100% methanol for 20 min at room temperature after antigen retrieval for 5 min at 121 °C using 10 mM citrate buffer (pH 6.0). Further, the sections were blocked using 10% goat serum for 30 min at RT. Further, incubation with primary antibody MCL-1 (Abcam, ab32087, 1:500) was achieved overnight at 4 °C. Further, the sections were washed using PBS. Sections were also incubated with biotinylated secondary antibody and horse radish peroxidase conjugated with streptavidin. Incubation with diaminobenzidine at RT for 2 min allowed formation of the brown color. The sections were further counter stained with hematoxylin. The slides were visualized using a light microscope.

### Anthropometric and biochemical

Venous blood from upper limb of the enrolled subjects were collected after fasting. Age, gender, body mass index (BMI), systolic blood pressure (SBP), diastolic blood pressure (DBP), blood lipid indexes such as total cholesterol, triglycerides, high-density lipoprotein cholesterol (HDL-C), and low-density lipoprotein cholesterol (LDL-C), albuminuria, eGFR, high-sensitivity C-reactive protein were all detected and recorded.

### Cell culture and hyperglycemic induction

Human renal proximal tubular cells (HK-2) were obtained from Bena Culture Collection, Suzhou, China. Cells were further cultured in DMEM (Gibco, Thermo Fisher) medium containing 10% FBS (Gibco), 100 U/mL penicillin, and 100 U/mL streptomycin. The cells were further maintained at 37 °C and 5% CO_2_. The cells were treated with normal glucose (5.5 mM d-glucose, NG group), high-glucose (30 mM d-glucose, HG group), or osmotic control conditions (5.5 mM glucose + 24.5 mM d-mannitol, OC group) for 12, 24 or 36 h. Further the cells were treated with CRD at 0–100 µM concentration for 1 h prior to incubation with NG or HG.

### Cell viability assay-MTT assay

HK-2 cells were initially seeded onto 96 well plates and post 24 h, cells were treated with varying concentrations of CRD. Further, cells were exposed to 10 µL of MTT. Finally, the purple formazan crystals formed were dissolved using DMSO and colorimetric assessment at 590 nm was performed.

### 5-Ethynyl-2′-deoxyuridine (EdU) staining assay

EdU staining of the cells were performed using BeyoClick™ EdU Cell Proliferation Kit with Alexa Fluor 488 (Beyotime). The cells were briefly treated with 10 µM EdU reagent for 2 h and counterstained with DAPI. The EdU stained cells were visualized and imaged for further assessment.

### Cell transfection

miR-193b-5p mimics and inhibitors and their respective controls were designed and synthesized by Gene Pharma (Shanghai, China). Cells were seeded onto a 24-well plate and were transfected with the mimics, inhibitors or the respective controls using lipofectamine 2000 reagent according to the manufacturer’s protocol.

### siRNA interference assay

Three pairs of small interfering RNAs (siRNAs) were designed to knockdown the expression of MCL-1. All siRNAs were synthesized by Ribobio (Guangzhou, China). The targeting sequences are listed followed: MCL-1#1-F: 5′-GCUUGUAAAUGUAUUUGUAAA-3′, MCL-1#1-R: 5′-UACAAAUACAUUUACAAGCUG-3′; MCL-1#2-F: 5′-GGUUACUGAUGACUUACAAAU-3′, MCL-1#1-R: 5′-UUGUAAGUCAUCAGUAACCUU-3′; MCL-1#1-F: 5′-GGGUUAGGACCAACUACAAAU-3′, MCL-1#3-R: 5′-UUGUAGUUGGUCCUAACCCUU-3′. The si-NC sequences were 5′-UUCUCCGAACGUGUCACGU-3′ (F) and 5′-ACGUGACACGUUCGGAGAAdTdT-3′ (R). siRNAs were transfected into HK-2 cells for 48 h using Lipofectamine 3000 (Invitrogen, Carlsbad, CA, USA), according to the manufacturer’s instructions.

### Flow cytometric assessment

Assessment of apoptosis was performed using Annexin V-FITC Apoptosis Detection Kit (#556570, BD Pharmingen). Cells were exposed to HG, miR inhibitors, mimics or CRD for 24 h and the cells were detached, centrifuged, washed and stained with 5 µL of Annexin V and 5 µL of PI with incubated at RT for 15 min in dark. Fluorescence was measured using flow cytometry and apoptosis levels were assessed.

The annexin-V/PI apoptosis assay kit (#556570, BD Pharmingen) was used according to the manufacturer’s recommendation to examine the apoptotic fraction of HK-2 cells. HK-2 cells were washed twice with PBS and resuspended in 100 µL of 1× binding buffer mixed with 5 µL of annexin-V-FITC and 5 µL of a PI staining solution for 15 min in the dark at room temperature. After 15 min of incubation, another 400 µL of binding buffer was added, and the cells were analyzed using a FACSCalibur flow cytometer (BD Biosciences, Franklin Lakes, NJ, USA). Ten thousand cells from the sample were scanned, and the data were analyzed using CellQuest software (BD Biosciences).

### TUNEL staining

To further assess level of apoptosis, we performed TUNEL staining using Cell Death Fluorescein Detection Kit (11684795910, Roche Molecular Biochemicals, Mannheim, Germany). Using the Olympus BX-51 light microscope, images were captured. TUNEL-positive cells were assessed from five images per section from each group. For the purpose of identifying apoptosis in renal cells, we employed a TUNEL staining kit (C10619, Invitrogen, USA). After slicing, renal tissues were deparaffinized with xylene twice for 5 min. Following rinsing in distilled water, the sections were dehydrated in a gradient of ethanol (100, 95, 80, 75, and 50%). The tissue sections were then treated in a proteinase K/10 mM Tris solution at 37 °C for 30 min. After applying TUNEL detection solution, each segment was incubated for 60 min at 37 °C in the dark. Anti-fluorescence quenching mounting reagent was used to seal the slices after they were washed three times in PBS. Finally, a fluorescent microscope was used to analyse the sections.

### Enzyme-linked immunosorbent assay

The enzyme-linked immunosorbent assay (ELISA) kits of inflammatory cytokine levels such as tumor necrosis factor alpha (TNF-α) and interleukin-6 (IL-6) were purchased from R&D Systems, Minneapolis, MN, USA.

### Assessment of oxidative stress

The assay kits of oxidative stress markers superoxide dismutase (SOD), phospholipid hydroperoxide glutathione peroxidase (GSH-Px), malondialdehyde (MDA) and catalase (CAT) in cell supernatant as well as kidney tissues were measured by commercially available kits, according to the manufacturer’s instructions (Nanjing Jiancheng Bioengineering Institute, Nanjing, China).

### PAS and Masson staining

Renal tissue sections at 4 μm were subjected to Periodic acid Schiff (PAS) and Masson’s trichrome staining to assess glycogen and collagen deposition, respectively. Assessment of glomerular mesangial dilation and sclerosis were performed using previously published protocol [23].

### MitoSox staining

MitoSox red (Thermo Fisher Scientific, Waltham, MA, USA) was used to assess mitochondrial ROS levels in HK-2 cells, based on the manufacturer’s instructions. Initially, cells were cultured in 24 well plate for 48 h and subsequently incubated with MitoSox red at a final concentration of 5 µM at 37 °C for 30 min. Finally, cells were washed thrice with PBS before visualization under a light microscope.

### DCFH-DA staining

ROS levels were also assessed by staining the cells with dichloro-dihydro-fluorescein diacetate (DCFH-DA, Beyotime Biotechnology) and measured by flow cytometry. HK-2 cells were briefly cultured for 48 h in a 6 well plate. Further, 10 µM of DCFH-DA dye was added to the cells and incubated for 20 min at 37 °C in dark. Post washing the cells with PBS, fluorescent images were obtained at 488/525 excitation/emission wavelengths.

### JC-1 staining

JC-1 fluorescence staining was performed to assess the membrane potential of HK-2 cells. Initially, the cells were incubated with JC-1 (10 µg/mL) for 20 min in dark at 37 °C. Post wash, the cells were immediately and visualized using a light microscope.

### Dual-luciferase assay

HK-2 cells were co-transfected with wt or mut MCL-1 3′UTR dual-luciferase reporter (100 ng) with miR-193b-5p mimic or NC (50 nM) by using Lipofectamine 3000 reagent (Invitrogen, Carlsbad, CA, USA) in 96-well plates based on the manufacturer’s instructions. The activities of luciferase were measured using Dual-luciferase reporter assay system according to the manufacturer’s instructions (Promega) after 36 h transfection. Firefly luciferase activity was normalized to renilla luciferase activity to adjust for transfection efficiency. Data are expressed as relative luciferase activities.

### Western blot

HK-2 cells were thoroughly lysed using RIPA lysis buffer. Total protein in the lysate was assessed using Bradford assay. Equal amounts of proteins were loaded onto sodium dodecyl sulphate polyacrylamide gel. The separated samples were then transferred onto a PVDF membrane. The membrane was further blocked using 5% skim milk and incubated with primary antibodies (MCL-1, Abcam, ab32087, 1:1000), PCNA (Abcam, ab29, 1:1500), Cyclin D1 (Abcam, ab16663, 1:1000), BAX (Abcam, ab32503, 1:1000), Bcl-2 (Abcam, ab182858, 1:1000), Cleaved-caspase-3 (Cell signaling, #9664, 1:1500), NOX1 (Abcam, ab121009, 1:1000), NOX2 (Abcam, ab310337, 1:1500), SOD1 (Abcam, ab51254, 1:1000), SOD2 (Abcam, ab68155, 1:1000), Caspase-3 (Abcam, ab184787, 1:1000), β-Actin (Abcam, ab8226, 1:3000), and GAPDH (Abcam, ab8245, 1:2000), overnight at 4 °C. Further, the blots were washed and incubated with secondary antibody for 1 h at RT. Finally, the bands were visualized using ECL western blotting substrate (Invitrogen, 32109, USA). Western blot images were quantified by using ImageJ (V1.8.0.112, National Institutes of Health, Bethesda, MD), with the density of each band normalized to that of β-Actin or GAPDH (Additional file 2).

### Quantitative real-time PCR (qRT-PCR)

The total RNA was isolation using RNA isolation kit (Qiagen, Valencia, CA, USA). Reverse transcription of mRNA was carried out using cDNA reverse transcription kit (Applied Biosystems, Foster City, CA). qRT-PCR were carried out using the QuantiTect Probe RT-PCR Kit (QIAGEN, Valencia, CA, USA), based on the manufacturer’s instructions. The relative expression levels were calculated using the 2^−ΔΔCt^ method. GAPDH was used as the internal control. The primers used in the PCR reactions are listed in Table 2.

**Table 2:** Primer sequences used in this study

Gene	Froward (5′–3′)	Reverse (5′–3′)
MCL-1 (human)	GCGGTAATCGGACTCAACCT	CTCCCCTCCCCCTATCTCTC
MCL-1 (mouse)	GCCAGTCATCAGGCTAGTCA	TCAGAGGCCTCCCTTCACAT
TGF-β1(human)	GCAAGTGGACATCAACGGGT	TCCGTGGAGCTGAAGCAATA
HO-1 (human)	GAGCCTATGGCATCTTCCCC	GCTGCCACATTAGGGTGTCT
NOX1 (human)	AAGTGGATGGTCCCTTTGGC	TGCTGCATGACCAACCTTTT
NOX2 (human)	AAGTGCCCAAAGGTGTCCAA	CACAAGCATTGAACAGCCCC
SOD1 (human)	CTTCAGCGCTCTAGGTCAGG	CACAAACGGTCTCTGTTGCG
SOD2 (human)	GAGGTGGGGCAATAGAGAAG	TTCAGTGTGCTGCTGAGTTCT
PCNA (human)	TGTTGGAGGCACTCAAGGAC	TAGGTGTCGAAGCCCTCAGA
Cyclin D1 (human)	ATGCCAACCTCCTCAACGAC	GGACCTCCTTCTGCACACAT
Marcksl1(human)	GGCTACAGAGCCATCCACTC	TGACCTCACAAGGACAGCAC
Scyl3 (human)	CAGTTGGTGTTTGCAGAGCC	CTGGTGAGAGCAAGCAAGGA
Aak1 (human)	CCAGGGGCAGAAAGTTGGAT	AGACTGCACTGTGGGTTACG
Tpp1 (human)	GTAGAGGGCCAGGGTTTCTG	AGAAAGTTGGGCAGGGGTTG
Ankrd13a (human)	TGGGGCTGGAGGACAGATAA	AAGATTCCAGCGGGTTCCTG
Carf (human)	CTCAGGGGCAACTTGTGGAT	CTTCCAGCATTCCGGTCACT
Piga (human)	CCGTCTCAGCATGGCCTGTA	AGAGAGCTGGTAAATGTGGCTT
Stx16 (human)	GTAGCATTGCTGCGCTTGC	CACTTAGGAGGTGGCCGTTT
miR-193b-5p	CGGGGTTTTGAGGGCG	AGTGCAGGGTCCGAGGTATT
GAPDH (human)	TTGCCCTCAACGACCACTTT	CAAGAAAGTTGGGTACTCCTTG
GAPDH (mouse)	CTACCCCCAATGTGTCCGTC	TGAAGTCGCAGGAGACAACC
U6	CTCGCTTCGGCAGCACA	AAACGCTTCACGAATTTGCGT

### Statistical analysis

Data are provided as mean ± standard deviation (SD) of at least three experimental repeats performed independently. All analysis were performed using Graphpad Prism 8.0 software (GraphPad software Inc). Differences among groups were carried out by the one-way ANOVA followed by the Bonferroni post hoc test. Pearson’s correlation coefficients were calculated for correlation analysis. *P* < 0.05 was considered statistically significant.

## Results

### miR-193‑5p was downregulated while MCL-1 was up-regulated in DN patients and in vitro HG-induced HK-2 cells

**Fig. 1:**
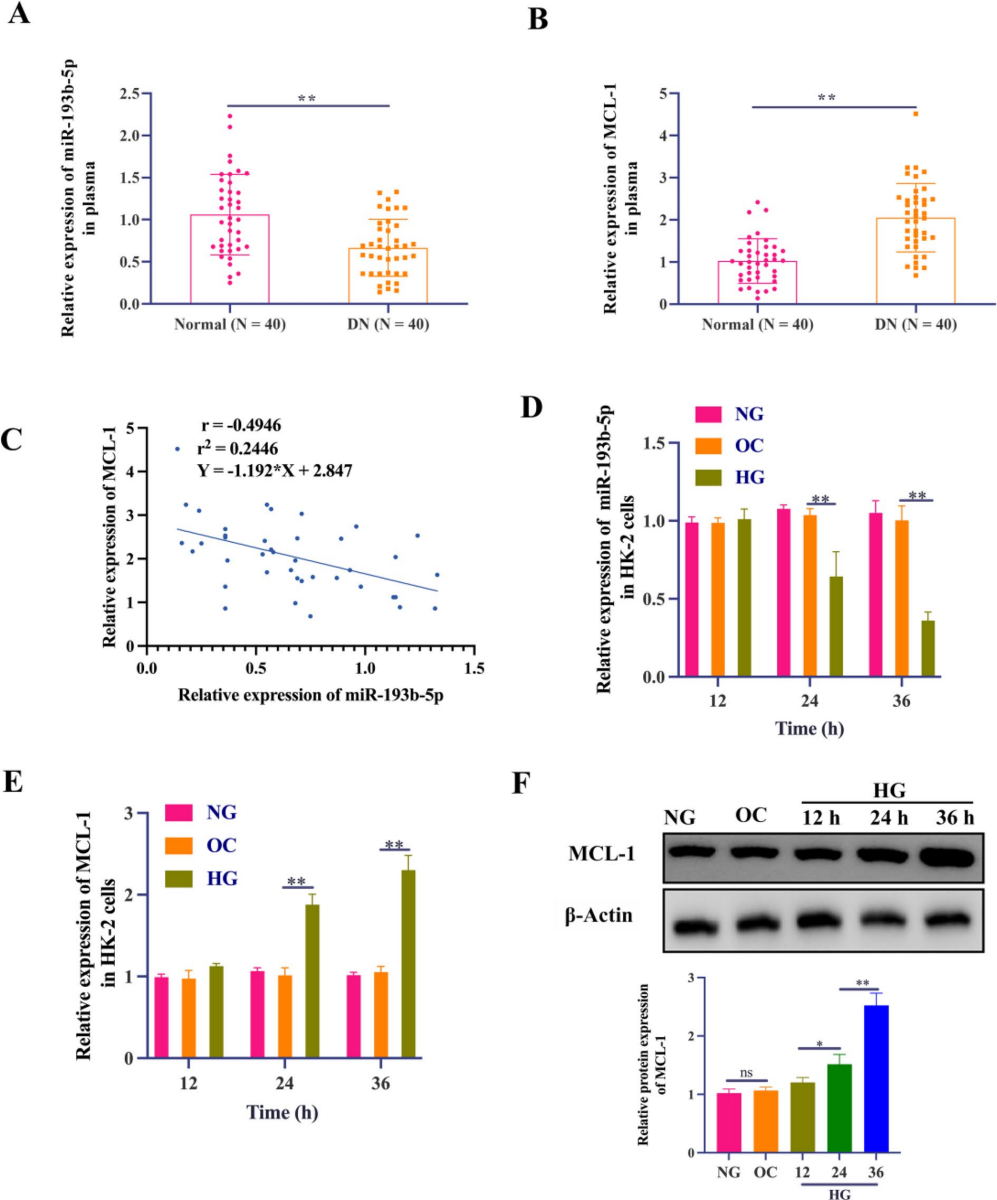
miR-193‑5p was downregulated while MCL-1 was up-regulated in DN patients and in vitro HG-induced HK-2 cells. **A**, **B** The expression of miR-193b-5p and MCL-1 in the serum of DN patients with microalbuminuria or macroalbuminuria and normal healthy volunteers was measured by qRT-PCR, n = 40. **C** An inverse correlation between *MCL-1* mRNA expression and miR-193b-5p expression was observed in the serum samples of DN patients with microalbuminuria or macroalbuminuria, n = 40. **D**–**F** HK-2 cells were treated with NG, OC, and HG for 12, 24, and 36 h, respectively. **D** qRT-PCR was used to evaluate the levels of miR-193b-5p in HK-2 cell, n = 6, U6 was used as internal control. **E**, **F** qRT-PCR (n = 6, GAPDH, internal control) and Western Blotting analysis (n = 3, β-Actin, internal control) were used to evaluate mRNA and protein expression of MCL-1 in HK-2 cells. Data are expressed as mean ± SD, *P < 0.05, **P < 0.01

Initially, using the GEO2R analysis tool (http://www.ncbi.nlm.nih.gov/geo/geo2r/), we understood the role of miRs in DN by performing the microarray analysis from GEO dataset (GEO121221) on the mid-morning urine samples from DN patients. Heatmapping and volcano plot of the array data indicated miR-193b-5p is potentially downregulated in DN patients (Additional file 1: Fig. S1). Additionally, qRT-PCR was utilized to demonstrate that levels of miR-193b-5p are lower, whereas levels of MCL-1 expression are higher, in the plasma of DN patients with micro or macroalbuminuria compared to normal healthy volunteers (Fig. 1A, B). Additionally, Pearson’s correlation analysis indicated a negative correlation between miR-193b-5p and *MCL-1* mRNA expression in DN patients (Fig. 1C). We used HK-2 cell line to further assess the effects of glucose levels on DN. We initially confirmed that indeed in the presence of HG levels, within 24 h, the cells express relatively low levels of miR-193b-5p, when compared with respective control cells (Fig. 1D). Alternatively, cells express high levels of MCL-1 within 24 h of exposure to HG (Fig. 1E, F).

### CRD treatment reversed the effects of HG on cell viability and apoptosis in HK-2 cells

**Fig. 2:**
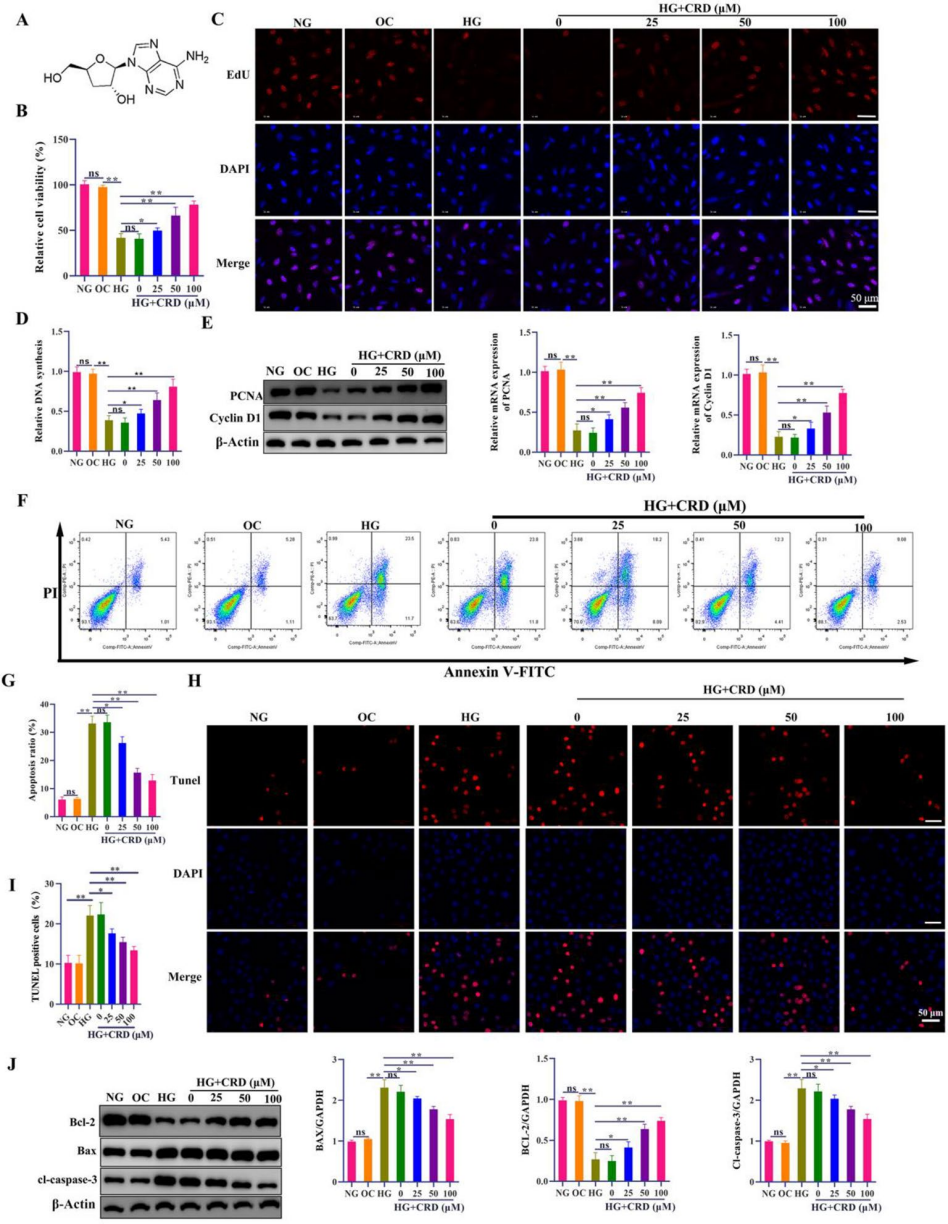
CRD treatment reversed the effects of HG on cell viability and apoptosis in HK-2 cells. HK‑2 cells were incubated with NG, OC or HG for 24 h. HG-cultured HK-2 cells were pre-incubated with different doses of CRD (0, 20, 50, and 100 µM) for 1 h. **A** The chemical structural formula of CRD. **B** Cell viability was determined using MTT, n = 6. **C**, **D** The proliferative ability of HK-2 cells was examined by EdU staining assay, bar, 50 μm, n = 3. **E** Western blotting analysis was performed to analyze the protein expression of PCNA and cyclin D1 (n = 3, β-Actin, loading control). **F, G** Flow cytometry analysis and quantitative analysis of total apoptotic cells, n = 5. **H**, **I** TUNEL assay determined level of apoptosis of HK-2 cells, scale bar, 50 μm, n = 3. **J** Western blot analysis of apoptosis related proteins (Bcl-2, BAX, and Cl-Caspase-3) (n = 3, β-Actin, loading control). Data are expressed as mean ± SD, ns, no significance, *P < 0.05, **P < 0.01

The apoptosis of renal tubular epithelial cells caused by HG is one of the key mechanisms linked to the onset of DN [24]. We were further interested to assess the effect of potential target drug, CRD, at varying concentrations (25, 50 and 100 µM) on HG induced decrease in viability (Fig. 2A). In our in vitro model, we treated HK-2 cells with HG for 24 h, and observed a significant decrease in viability of cells, as indicated by MTT assay. Indeed, treatment with CRD significantly reversed the decrease in cell viability caused by exposure to HG (Fig. 2B). At 100 µM of CRD, we could observe the maximum rescue of cell viability. Additionally, we performed EDU staining, and the number of EDU^+^ cells post exposure to HG was significantly lowered. However, treatment with CRD (25, 50 and 100 µM) significantly rescued and increased the number of EDU^+^ cells in cells exposed to HG (Fig. 2C, D). Cyclin D1 and PCNA levels were also assessed as they are vital regulators of cell cycle progression. These findings confirmed our earlier findings that exposure to HG dramatically reduced cyclin D1 and PCNA levels. Exposure to CRD, however, counteracted this effect of HG (Fig. 2E). Apoptotic cell rate was detected by flow cytometric analysis of Annexin V-FITC/PI staining, which indicated a significant increase in apoptotic cells post exposure to HG. However, exposure to CRD (25–100 µM) significantly decreased the number of apoptotic cells (Fig. 2F, G). Similarly, TUNEL staining also indicated a significant increase in apoptosis post exposure to HG, which could be rescued by exposure to CRD (25, 50 and 100 µM) (Fig. 2H, I). We also saw an interesting decrease in Bcl-2 levels and a large increase in Bax and cleaved caspase-3 levels when HK-2 cells were exposed to HG. Nevertheless, treatment with various CRD doses resulted in a considerable reduction in Bax and cleaved caspase-3 levels and an increase in Bcl-2 levels (Fig. 2J).

### CRD treatment relieve HG-induced HK-2 cell damage by inhibiting oxidative stress and abnormal energy metabolism

**Fig. 3:**
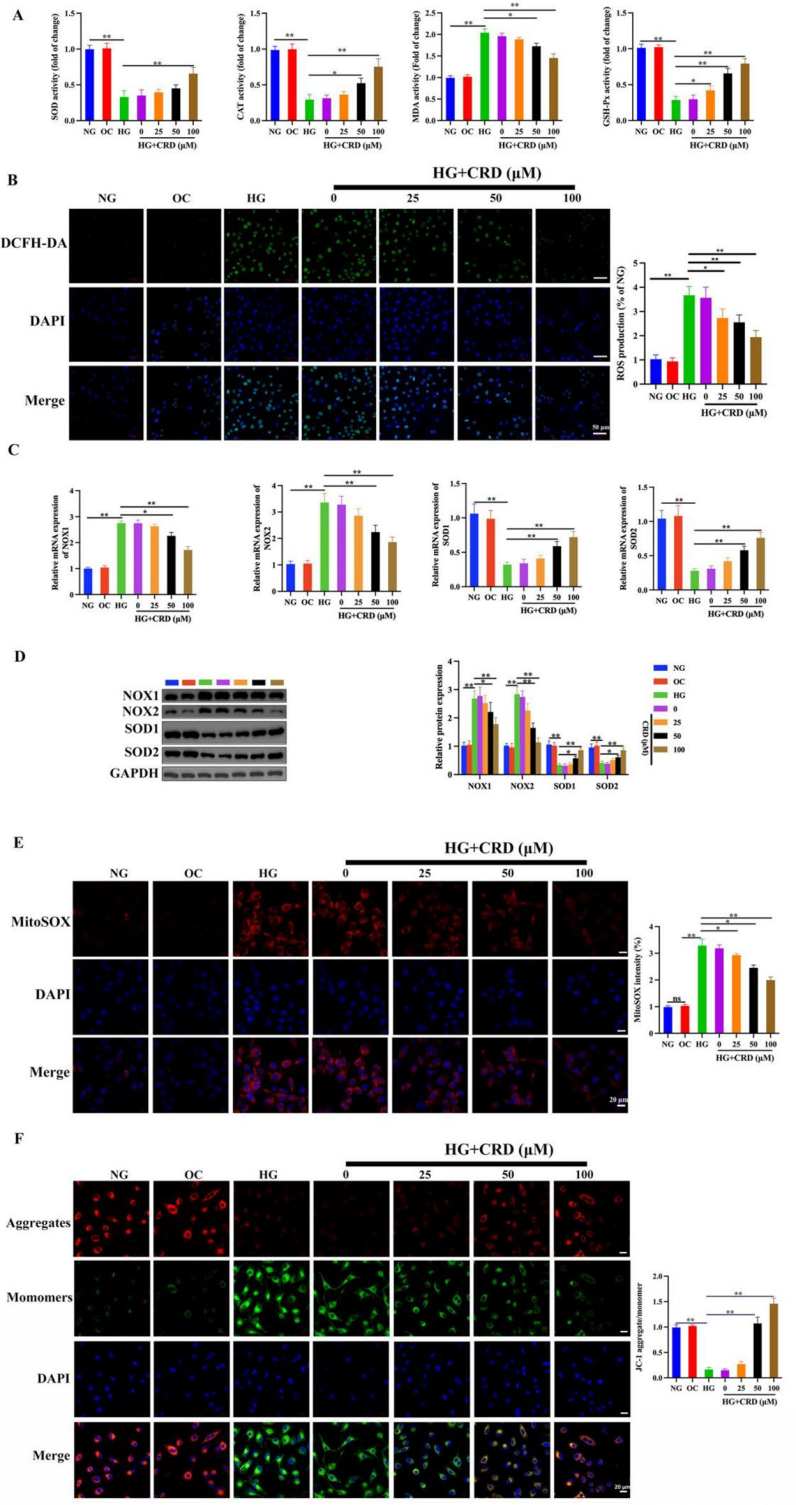
CRD treatment relieve HG-induced HK-2 cell damage by inhibiting oxidative stress and abnormal energy metabolism. HK‑2 cells were incubated with NG, OC or HG for 36 h. HG-cultured HK-2 cells were pre-incubated with different dose of CRD (0, 20, 50, and 100 µM) for 1 h. **A** The activities of antioxidant enzymes (SOD, MDA, CAT and GSH-Px) were evaluated, n = 5. **B** Intracellular ROS level of HK-2 cells was verified by DCFH-DA staining, scale bar = 50 μm, n = 3. **C**, **D** The mRNA and protein expression of oxidative stress indicators (NOX1, NOX2, SOD1, and SOD2) in HK-2 cells was detected by qRT-PCR (n = 6, GAPDH, loading control) and western blot analysis (n = 3, GAPDH, loading control). **E** MitoSOX staining was performed to asses mitochondrial ROS levels, scale bar = 50 μm, n = 3. **F** The mitochondrial membrane potential was measured by JC-1 staining, Red (JC-1 aggregate)/green (JC-1 monomer) fluorescence intensity represents the potential alteration of mitochondria membrane, scale bar = 50 μm, n = 3. Data are expressed as mean ± SD, *P < 0.05, **P < 0.01

We evaluated important oxidative stress markers to better understand the molecular mechanisms via which CRD could potentially mitigate HG induced HK-2 cell damage. When we initially treated the cells with HG, we observed a significant decrease in antioxidant enzymes such as SOD, GSH-Px and CAT. However, treatment with CRD reversed this effect and recovered the antioxidant enzyme levels (Fig. 3A). Additionally, we observed significantly high levels of MDA, a key indicator of increase in free radicals, post exposure of cells to HG. Exposure to CRD could significantly reverse this effect as well (Fig. 3A). We also assessed the intracellular ROS activity using DCFH-DA staining. Evidentially, exposure of HK-2 cells to HG significantly increased ROS activity, however, treatment with CRD significantly reversed this effect (Fig. 3B). In diabetic individuals, hyperglycemia and insulin resistance are related with the aetiology of DN. It is believed that abnormal lipid metabolism, inflammation, and oxidative stress are the root causes of the condition [25]. In order to give further evidence, we then determined the levels of mRNA and protein expression of oxidative stress markers (NOX1, NOX2, SOD1, and SOD2) in each of the different treatment cohorts. After being exposed to HG, the levels of oxidase indicators (NOX1, NOX2) were found to significantly increase, whereas the levels of antioxidant enzyme markers (SOD1 and SOD2) significantly decreased. However, it was discovered that this effect may be undone through the utilisation of CRD (Fig. 3C, D). We further performed MitoSOX staining studies to assess the levels of mitochondrial ROS levels. Indeed, exposure to HG significantly increased mitochondrial ROS levels, whereas CRD treatment decreased the ROS levels (Fig. 3E). JC-1 staining of the cells also indicated a decrease in the mitochondrial membrane potential in cells exposed to HG, however treatment with CRD led to a significant rescue of the mitochondrial membrane potential (Fig. 3F). Taking all these data together, it is evident that CRD potentially rescues the HG-induced HK-2 cell damage through inhibition of oxidative stress and by regulating the energy metabolism.

### CRD treatment reverses miR-193b-5p/MCL-1 expression changes induced by HG in HK-2 cells

**Fig. 4:**
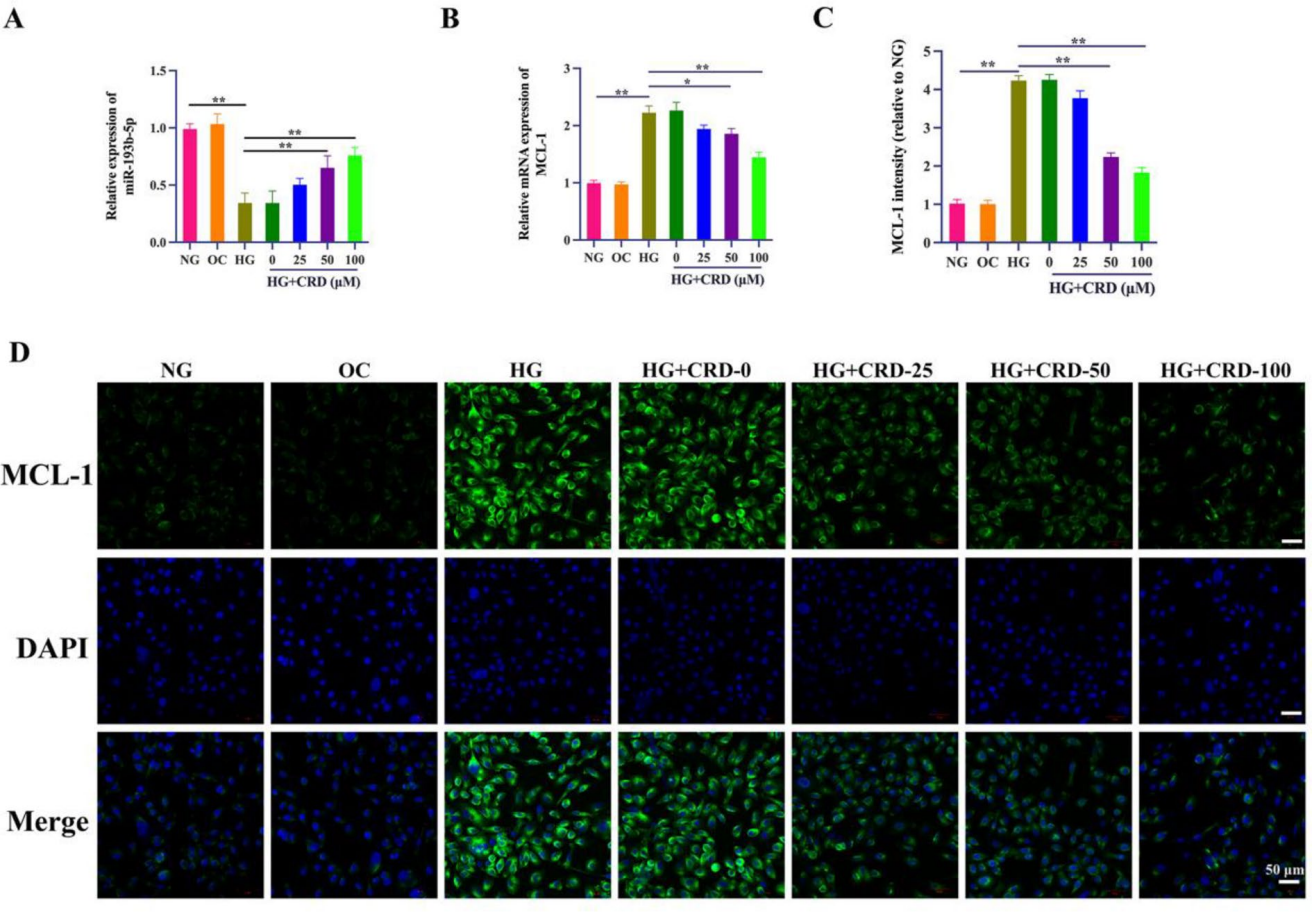
CRD treatment reverses miR-193b-5p/MCL-1 expression changes induced by HG in HK-2 cells. HK‑2 cells were incubated with NG, OC or HG for 36 h. HG-cultured HK-2 cells were pre-incubated with different dose of CRD (0, 20, 50, and 100 µM) for 1 h. **A** The expression of miR-193b-5p in different culture was measured by qRT-PCR (n = 6, U6, loading control). **B** The mRNA expression of MCL-1 was detected by qRT-PCR (n = 3, GAPDH, loading control). **C**, **D** Immunofluorescence staining of MCL-1 (green) in HK-2 cells. Nuclei were counterstained with DAPI (blue), scale bar = 50 μm, n = 3. Data are expressed as mean ± SD, *P < 0.05, **P < 0.01

When cells were treated with CRD and exposed to HG for 36 h, the expression of miR-193b-5p was noticeably higher than in the control HG samples (Fig. 4A). Similarly, we observed significantly lower *MCL-1* mRNA expression in cells rescued by CRD, when compared with control samples (Fig. 4B). We also observed MCL-1 protein expression levels to be high in cells exposed to HG which could be significantly reversed by treatment with CRD, as indicated by both western blotting and immunofluorescence assays (Fig. 4C, D).

### MiR-193b-5p negatively regulated MCL-1 in HK-2 cells cultured in HG

**Fig. 5:**
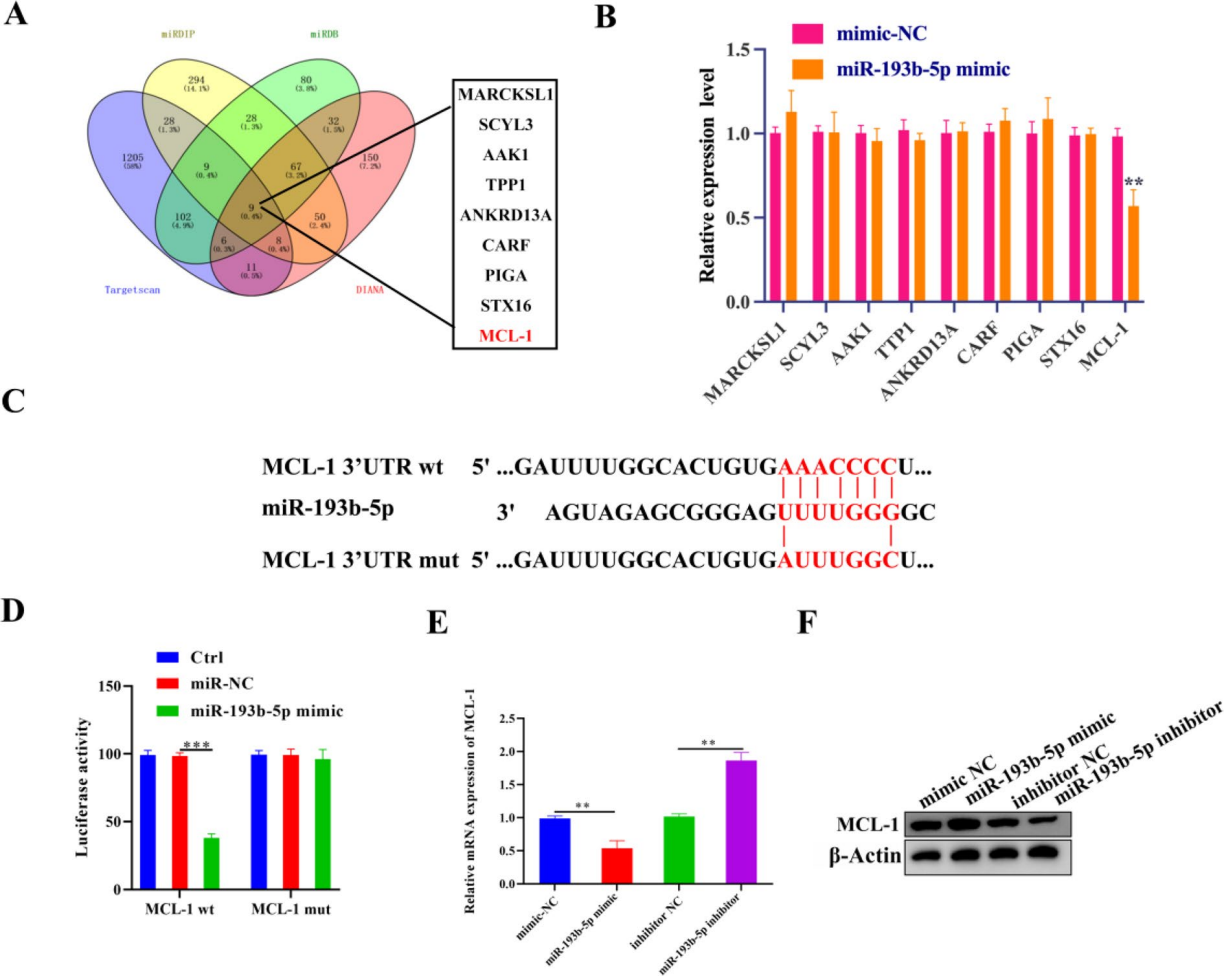
MiR-193b-5p negatively regulated MCL-1 in HG-cultured HK-2 cells. **A** TargetScan, miRDB, DIANA, and miRDIP each identified nine mRNAs as miR-193b-5p downstream genes, with MCL-1 being one of them. **B** qRT-PCR was used to explore the effect of miR-193b-5p overexpression on expression levels of candidate mRNAs, (n = 6, U6, loading control). **C** Targetscan (https://www.targetscan.org/vert_80/) was used to evaluate the miR-193b-5p binding site on 3′-UTR of MCL-1. **D** The relative luciferase activity was determined in HK-2 cells 48 h after transfection with the miR-193b-5p mimic/mimic NC/Ctrl or the 3′UTR of MCL-1 wt/mut constructs, n = 6. **E**, **F** The expression of MCL-1 in HK-2 cultured in HG were determined using qRT-PCR (n = 6, GAPDH, loading control) and western blotting (n = 3, β-Actin, loading control) after transfection with miR-193b-5p mimic/mimic NC or miR-193b-5p inhibitor/inhibitor NC for 48 h, β-Actin was used as internal control. Data are expressed as mean ± SD, **P < 0.01

We evaluated miR-193b-5p’s downstream targets with additional bioinformatics instruments, including TargetScan, miRDB, DIANA, and miRDIP (Fig. 5A). Based on this evaluation, it was clear that MCL-1 was one of the top ten genes controlled by miR-193b-5p. We demonstrated that MCL-1 expression was indeed considerably downregulated in the presence of miR-193b-5p mimics (Fig. 5B). TargetScan evaluation also revealed that miR-193b-5p binds to the 3′ UTR of MCL-1 (Fig. 5C). In addition, we performed luciferase assays with 3′ UTR-mutated MCL-1. Similar to prior findings, we detected a substantial reduction in luciferase activity when miR-193b-5p mimics were introduced to cells expressing MCL-1 wild type (Fig. 5C). In contrast, when cells expressing mutant MCL-1 were exposed to miR-193b-5p mimics, no significant variation in luciferase activity was found when compared to respective controls (Fig. 5D). These findings suggest that miR-193b-5p binds to the 3′ UTR region of MCL-1. We observed a significant decrease in MCL-1 mRNA expression after cells were treated with miR-193b-5p mimics. However, when cells were treated with a miR-193b-5p inhibitor, *MCL-1* mRNA levels considerably increased (Fig. 5E). Using western blotting analysis, we could replicate these findings at the protein expression level (Fig. 5F). These results demonstrated that miR-193b-5p binds to MCL-1 and modulates its expression.

### Protective effect of CRD against HG challenge to HK-2 cells is mediated through the regulation of expression of miR-193b-5p/MCL-1 axis

**Fig. 6:**
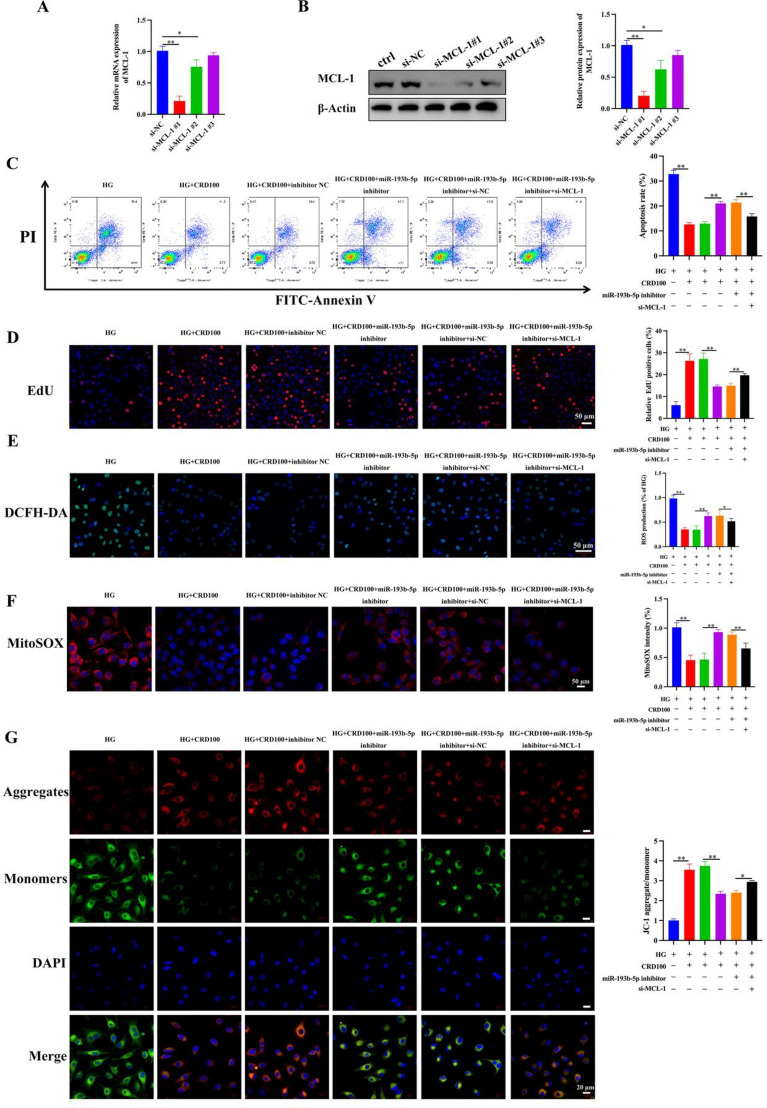
Protective effect of CRD against HG challenge to HK-2 cells is mediated through the regulation of expression of miR-193b-5p/MCL-1 axis. HG-cultured HK-2 cells were transfected with inhibitor NC or miR-193b-5p inhibitor, along with si-MCL-1 or si-NC in the presence of CRD (100 µM, CRD100). **A**, **B** Detection of mRNA and protein expression of MCL-1 utilizing qRT-PCR (n = 6, GAPDH, loading control) and western blotting (n = 3, β-Actin, loading control). **C** Flow cytometry analysis and quantification of cell apoptosis in HK-2 cells in different groups at 48 h, n = 6. **D** The proliferative ability in different groups were detected by EdU staining assay, scale bar = 50 μm, n = 3. **E** ROS level of HK-2 cells was verified by using DCFH-DA assay, scale bar = 50 μm, n = 3. **F** MitoSOX staining for mitochondrial ROS scale bar = 50 μm, n = 3. **G** The mitochondrial membrane potential was measured by JC-1 staining, Red (JC-1 aggregate)/green (JC-1 monomer) fluorescence intensity represents the potential alteration of mitochondria membrane, scale bar = 50 μm, n = 3. Data are expressed as mean ± SD, *P < 0.05, **P < 0.01

Further, in order to comprehend the molecular mechanism by which CRD regulates effects against HG, we inhibited MCL-1 and evaluated decreased mRNA and protein expression (Fig. 6A, B). We did additional flow cytometric examination of cells to evaluate apoptosis, and consistent with earlier findings, exposure to HG raised apoptosis levels, whereas CRD therapy greatly restored the cells. Application of miR-193b-5p inhibitors, however, reversed this result and increased the frequency of CRD-induced apoptotic cells. Furthermore, inhibiting MCL-1 once more decreased the number of apoptotic cells, indicating that miR-198b-5p controlled apoptosis through MCL-1 (Fig. 6C). EdU staining revealed that exposure to HG considerably lowered proliferation, but treatment with CRD significantly enhanced proliferation and reversed this effect. In contrast, suppression of miR-193b-5p dramatically inhibited cell proliferation, but silencing MCL-1 restored the cells and boosted cell proliferation (Fig. 6D). The DCFH-DA assay revealed that cells exposed to HG had elevated ROS levels, which could be reduced by CRD therapy. Similar to apoptosis and proliferation, suppression of miR-193b-5p enhanced ROS levels, which was reversed by silencing MCL-1 (Fig. 6E). MitoSOX and JC-1 staining were utilized to evaluate the effects on mitochondria. MitoSOX staining revealed that exposure to HG dramatically elevated mitochondrial ROS levels, which could be significantly reduced by exposure to CRD (Fig. 6F). However, suppression of miR-193b-5p restored this impact, and silencing of MCL-1 once again recovered the cells. These outcomes were likewise observed with JC-1 staining (Fig. 6G). All of the aforementioned findings show that CRD protects cells from HG-induced stress via modulating the miR-193b-5-/MCL-1 axis.

### CRD mediates renal function parameters in DN mice by regulating miR-193b-5p expression

**Fig. 7:**
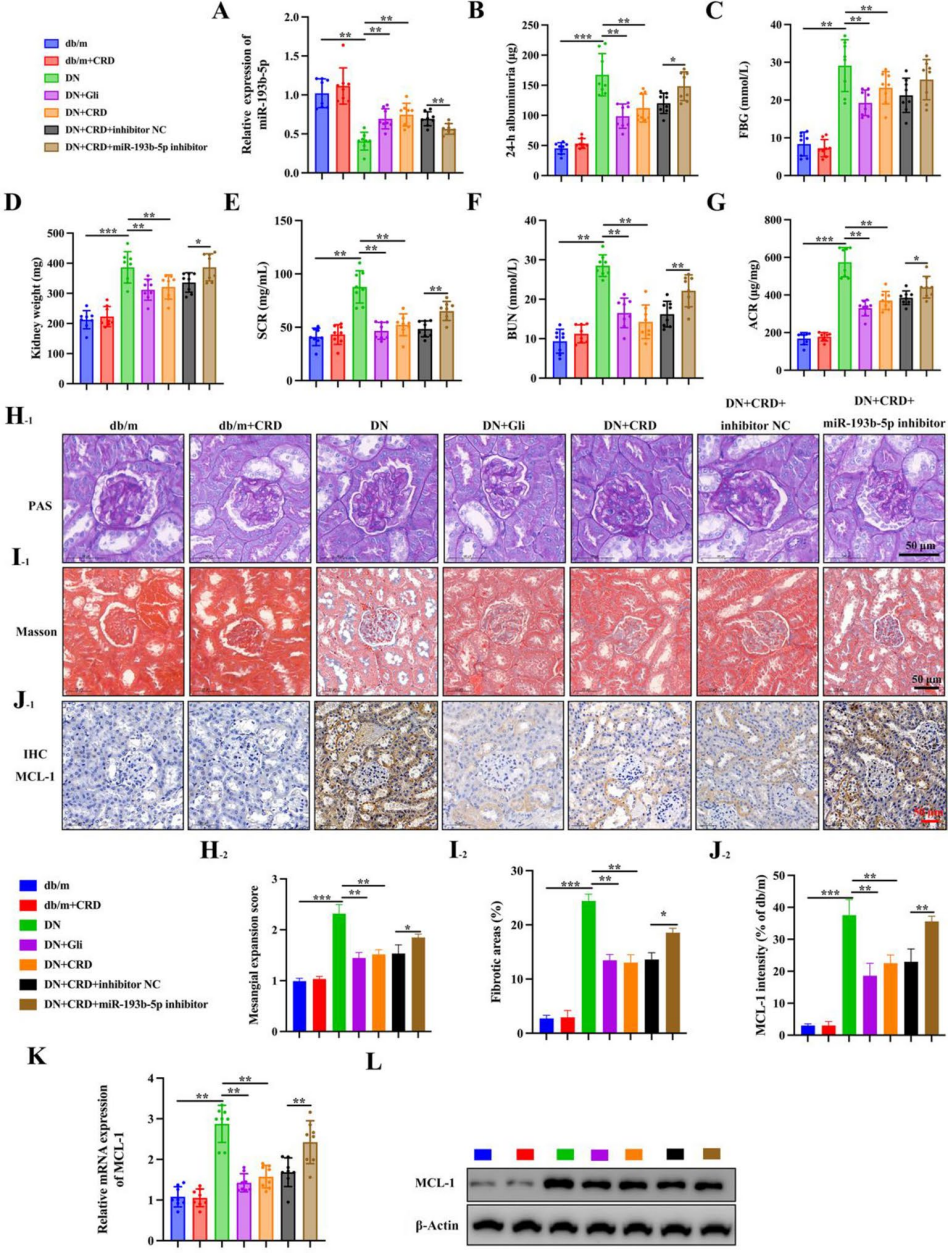
CRD mediates renal function parameters in DN mice by regulating miR-193b-5p expression.** A** The expression of miR-193b-5p in kidney tissues was evaluated by qRT-PCR, U6 was used as internal control, n = 8. **B**–**D** 24 h urine albumin level, kidney weight (KW), and fasting blood glucose (FBG) were assessed after 12 weeks of CRD treatment, n = 8. **E**–**G** The serum creatinine (SCR), blood urea nitrogen (BUN), urine albumin/creatinine ratio (ACR) of different groups, n = 8. **H**, **I** Kidney tissues from different groups were stained with PAS and Masson staining, scale bar, 50 μm. Mesangial expansion and fibrotic areas were measured, n = 5. **J** Immunohistochemical staining analysis of MCL-1, scale bar, 50 μm, n = 5. **K** The mRNA expression of *MCL-1* was evaluated by qRT-PCR, GAPDH was used as internal control, n = 8. **L** Relative expression of MCL-1 in renal tissue were determined using western blotting analysis, n = 5, β-Actin was used as internal control. Data are expressed as mean ± SD, *P < 0.05, **P < 0.01, ***P < 0.001

CRD was delivered to db/db mice to test its influence on DN. Furthermore, we examined the expression of miR-193b-5p in renal tissues and discovered that DN animals had significantly lower levels of miR-193b-5p than normal mice. We detected a significant rise in miR-193b-5p levels when these mice were treated with CRD at several concentrations (Fig. 7A). DN mice had dramatically elevated 24 h urine albumin, FBG, and KW levels. However, after 12 weeks of treatment with CRD, these parameters were dramatically lowered, but suppression of miR-193b-5p reversed CRD’s protective effect (Fig. 7B–D). Similarly, we evaluated serum creatinine (SCR), blood urea nitrogen (BUN), and urine albumin/creatinine ratio (ACR) of the various groups and found that DN mice had elevated levels of SCR, BUN, and ACR, whereas exposure to CRD drastically decreased these levels. However, the usage of miR-193b-5p significantly increased the levels of the previously described markers (Fig. 7E–G). With PAS and Masson’s staining, kidney tissues from separate groups were stained. Evidently, the mesangial expansion score and fibrotic area were considerably increased in DN kidney sections, but the expansion score and fibrotic area were decreased in CRD-treated animals. The miR-193-3p inhibitor greatly lowered this CRD-mediated rescue (Fig. 7H, I). MCL-1 immunohistochemistry staining on renal tissue slices from mice with DN revealed an increase in staining intensity; however, CRD therapy corrected this effect. In contrast, suppression of miR-193b-5p dramatically boosted MCL-1 staining levels and diminished the effect of CRD (Fig. 7J). In addition, we verified these results by measuring mRNA and MCL-1 protein levels (Fig. 7K, L).

### CRD modulates oxidative stress and inflammation in DN mice by regulating miR-193b-5p expression

**Fig. 8:**
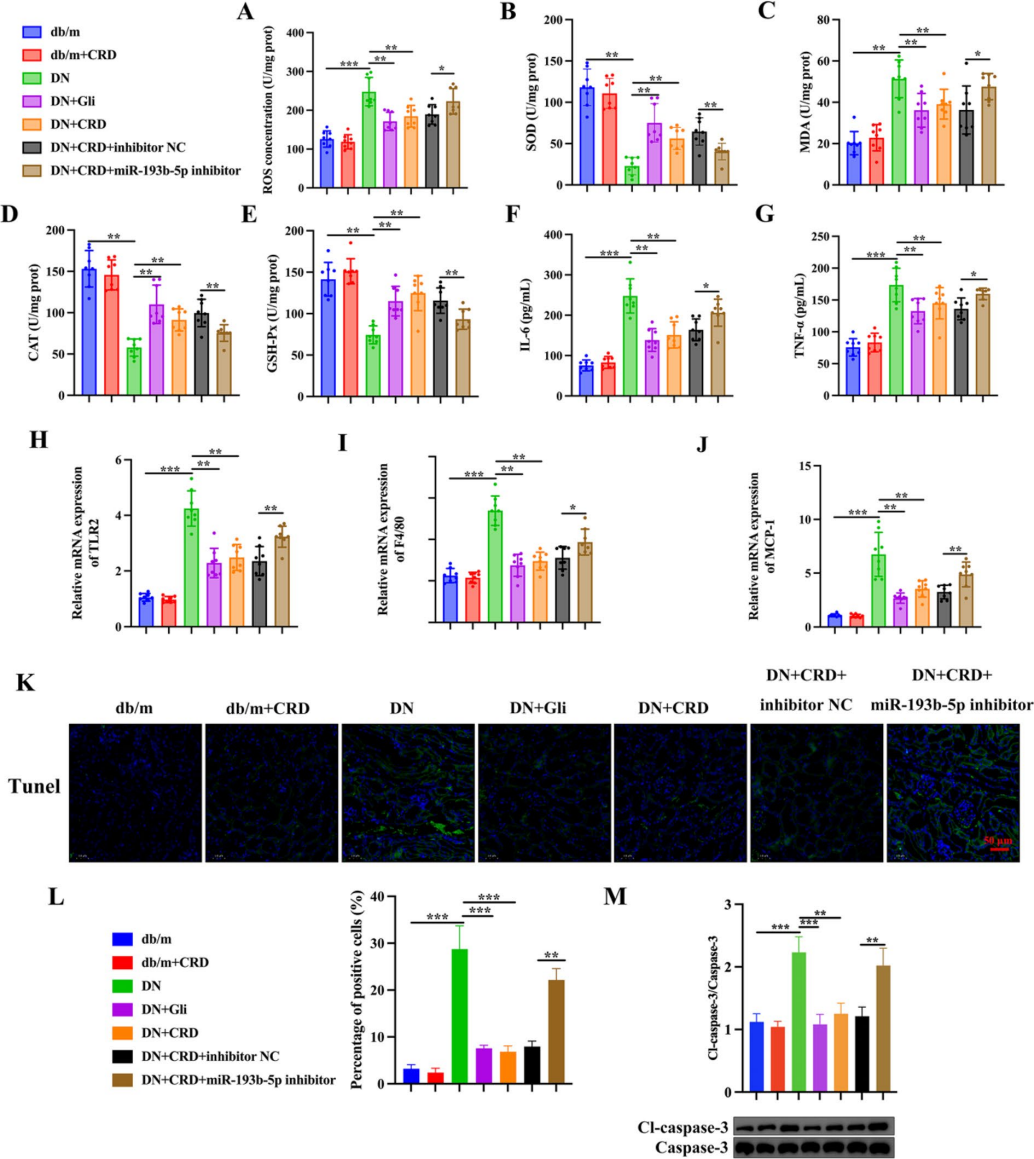
CRD modulates oxidative stress and inflammation in DN mice by regulating miR-193b-5p expression. **A**–**E** The levels of ROS, MDA, SOD, CAT and GSH-Px in renal tissues form different groups of mice were detected by ELISA, n = 8. **F**, **G** Measurement of IL-6 and TNF-α in serum of mice form different group were detected by ELISA assays, n = 8. **H**–**J** The expression of proinflammatory genes (TLR2, F4/80, and MCP-1) in renal tissues from different group were detected by qRT-PCR analysis, GAPDH was used as internal control, n = 8. **K**, **L** Representative kidney TUNEL staining of different groups, n = 5. Scale bar, 50 μm. **M** Western blots for Cleaved-caspase-3 and Caspase-3 protein expressions in kidney tissue extracts, n = 5. Data are expressed as mean ± SD, *P < 0.05, **P < 0.01, ***P < 0.001

In the renal tissues of DN mice, an increase in ROS and MDA levels, as well as a decrease in SOD, CAT, and GSH-Px levels, indicated an increase in oxidative stress. In contrast, treatment with CRD lowered ROS and MDA levels while boosting SOD, CAT, and GSH-Px levels. miR-193b-5p inhibition dramatically altered the protective effect of CRD against oxidative stress (Fig. 8A–E). Additionally, we found similar elevations in inflammatory markers (IL-6 and TNF-α) and pro-inflammatory genes in the serum of DN mice, including TLR2, F4/80, and MCP-1. Similar to ROS levels, CRD therapy significantly reduced inflammatory markers. However, miR-193b-5p inhibition caused the opposite effect (Fig. 8F, G). Moreover, TUNEL labeling revealed a considerable increase in apoptosis in the kidney tissues of DN mice. However, CRD administration greatly reduced apoptosis levels, which was reversed by treatment with miR-193b-5p inhibitor (Fig. 8K, L). Western blot analysis of cleaved-caspase 3/Caspase-3 protein expressions validated these findings further (Fig. 8M).

## Discussion

DN is a microvascular consequence of diabetes that can be very dangerous and is the largest cause of end-stage renal failure worldwide [26, 27]. Given the severe complications and heavy health burden associated with DN, there is an urgent need to develop novel early interventions to halt the progression of this debilitating microvascular complication of diabetes. *C. militaris* has been shown to have many beneficial effects ranging from anti-inflammation [28], anti-diabetic [29], along with being anti-infectious [30]. Studies have also indicated a potential anti-tumorous activity [31]. CRD is derived from *C. militaris*, a fungi prevalently used in Asian traditional medicine [32]. CRD, being a natural medication, may offer certain advantages in terms of safety and clinical compliance when compared to traditional therapy for DN. Recent research has revealed that CRD exhibits therapeutic properties in rats with DN through the stimulation of autophagy [15]. The results of this study show that CRD is capable of reversing the decreased expression of miR-193b-5p that is caused by DN and, as a result, is responsible for mediating renal function parameters in DN mice. The underlying mechanism of action concerning CRD relieving DN was elucidated and the miR-193b-5p was identified as a promising target in DN, contributing to the popularized use of CRD and novel therapies development for DN.

The exploration of the potential therapeutic applications of Traditional Chinese medicine (TCM) in modulating miRNAs for the treatment of diseases is a promising avenue for future research. By further elucidating the underlying mechanisms of miRNAs action, particularly in the context of DN therapy, a solid theoretical foundation may be established to support diverse clinical therapeutic approaches [33]. Numerous studies have provided evidence suggesting that there is dysregulation of miRNAs throughout the evolution of DN. For example, it has been documented that miRNA-21 plays a crucial role as a pathogenic component in DN [34]. Endogenous miR-204 Protects the Kidney against Chronic Injury in Hypertension and Diabetes [35]. The scope of our research encompasses the utilisation of CRD in the therapy of DN, while also revealing a previously unknown method involving the modification of the miR-193b-5p/MCL-1 axis.

The progression of DN is intricately linked to heightened levels of oxidative stress, as well as modifications in renal metabolism [36]. Classical mechanisms clearly describe that HG could lead to an increase in oxidative stress which further contributes to proinflammatory markers [37, 38]. In this study, we discovered that miR-193b-5p is highly downregulated in DN. Indeed, in HG-exposed HK-2 cells, this downregulation was associated with decreased viability, increased inflammation, mitochondrial oxidative stress, and apoptosis. CRD treatment dramatically boosted miR-193b-5p levels and improved cell viability while decreasing inflammation, oxidative stress, and apoptosis. The role of miR-193b-5p in cancer is well known. miR-193b-5p is significantly downregulated in prostate cancer, and its overexpression appears to slow tumor growth by reducing the number of cells in the S phase of the cell cycle [39]. Similarly, overexpression of miR-193b-5p in esophageal cancer appears to trigger non-apoptotic cell death and autophagy [40]. According to one study, miR-193b-5p protects cells from acrylamide-induced toxicity and cell cycle arrest through modulating FoxO3, p21, and cyclin D1 [41]. Another study found that delivering miR-193b-5p to wounded brain via systemic exosomes decreased inflammation and enhanced healing in a subarachnoid hemorrhage mouse model [42]. These investigations clearly demonstrated miR-193b-5p’s potential cytoprotective and anti-inflammatory properties.

It is known that miRNAs regulate processes by activating or deactivating their downstream targets. miRNAs in DN have been found to have dual effects, miR-337, miR-93, miR-216a, and miR-21 has been identified to contribute to the progression of DN pathogenesis [43], while miR-342 and miR-25 [44] has been found to be protective against DN. Interestingly, miR-342 suppresses fibrosis in diabetic retinopathic kidneys by binding to the 3′UTR of SOX-6 and in turn inducing the degradation of SOX-6. Using bioinformatics tools, we identified that miR-193b-5p binds to MCL-1 and in turn downregulates its expression. Importantly, using a luciferase assay, we verified that miR-193b-5p binds to the 3′-UTR of MCL-1. CRD mediated overexpression of miR-193b-5p could drastically decrease MCL-1 levels and thus the cytotoxic effect of HG. We used miR-193b-5p inhibitors in the presence of CRD to establish that the miR-193b-5p/MCL-1 axis is indeed regulating this effect, and we noticed a complete reversal of CRD’s potency in rescuing cells from the detrimental effects of HG. These findings were supported by our work on db/db mouse models, in which we discovered that CRD can significantly lower MCL-1 levels and even protect the mice kidney from the effects of DN.

MCL-1 is a member of the antiapoptotic BCL2 family, and its significance in cancer is well understood. In the early stages of diabetes, MCL-1 deficiency appears to contribute to β cell death [45]. However, there are few research examining its function in the kidney during DN. We found that MCL-1 is considerably elevated in HG in this study. This is the first study to identify the miR-193b-5p/MCL-1 axis as a factor in the progression of DN. This research adds to the advancement of understanding and identification of new therapeutic targets, such as miR-193b-5p, for the creation of novel therapeutic techniques. Additionally, investigation of MCL-1’s effect on DN could aid comprehension of the complicated molecular pathways underlying DN. In addition, by this investigation, the effect of CRD on DN via the miR-193b-5p/MCL-1 axis has been identified and clarified.

## Conclusions

In conclusion, this study provides in vitro and in vivo evidence that CRD protects against diabetic DN. Based on our findings, it appears that CRD protects HK-2 cells from damage caused by HG by decreasing oxidative stress, inflammation, and apoptosis. Regulation of the miR-193b-5p/MCL-1 pathway mediates CRD’s nephroprotective effect. Renal function and pathology are both enhanced by CRD therapy in a mouse model of DN. Together, CRD’s ability to target the miR-193b-5p/MCL-1 axis and attenuate several harmful pathways suggests it has promise as a natural therapy for diabetic kidney disease. We need more research into the potential translational utility of CRD. In conclusion, our study elucidates hitherto unknown mechanisms of CRD in DN and highlights its therapeutic potential.

### Supplementary Information

**Additional file 1: Figure S1.** MiR-193b-5p is down-regulated in DN patient. **A** The cluster heatmap from microarray data (*P* < 0.05) (GEO121221) shows miRNAs in the mid-morning urine samples with different expression patterns among normal control and DN patients. Each column represents a sample, and each row represents a miRNA. miRNAs with a fold change ≥ 2 and *P* value ≤ 0.05 are shown in the heat map above. miR-193b-5p is marked with an arrow. **B **Volcano plot shows the up-regulated and down-regulated miRNAs in normal control and DN patient. miR-193b-5p is indicated with an arrow.

**Additional file 2.** Compiled set of unedited images of the original membrane used in the WB experiments.

## Data Availability

All datasets generated in this study are included in the article or in Additional files. The datasets used and/or analyzed during the current study are available from the corresponding author on reasonable request.

## Abbreviations

BMI: Body mass index
BUN: Blood urea nitrogen
CAT: Catalase
CRD: *Cordyceps militaris*
DN: Diabetic nephropathy
DM: Diabetes Mellitus
DBP: Diastolic blood pressure
DCFH-DA: Dichloro-dihydro-fluorescein diacetate
FBG: Fasting blood glucose
HDL-C: High-density lipoprotein cholestero
IL-6: Interleukin-6
KW: Kidney weight
LDL-C: Low-density lipoprotein cholesterol
MCL-1: Myeloid leukemia 1
miRNAs: MicroRNAs
MCL-1: Myeloid leukemia 1
qRT-PCR: Quantitative real-time PCR
GSH-Px: Phospholipid hydroperoxide glutathione peroxidase
PAS: Periodic acid Schiff
qRT-PCR: Quantitative real-time PCR
ROS: Reactive oxygen species
SCR: Serum creatinine
SOD: Superoxide dismutase
SBP: Systolic blood pressure
TCM: Tumor necrosis factor alpha
TNF-α: Traditional Chinese medicine
