# Supplementary material for: Cordycepin from Cordyceps militaris ameliorates diabetic nephropathy via the miR-193b-5p/MCL-1 axis
Source: Chin Med. 2023 Oct 13;18:134. doi: 10.1186/s13020-023-00842-5 (PMC10576278; doi:10.1186/s13020-023-00842-5)
Supplement: Supplementary file 1 — Additional file 1: Figure S1. MiR-193b-5p is down-regulated in DN patient. A The cluster heatmap from microarray data (P < 0.05) (GEO121221) shows miRNAs in the mid-morning urine samples with different expression patterns among normal control and DN patients. Each column represents a sample, and each row represents a miRNA. miRNAs with a fold change ≥ 2 and P value ≤ 0.05 are shown in the heat map above. miR-193b-5p is marked with an arrow. B Volcano plot shows the up-regulated and down-regulated miRNAs in normal control and DN patient. miR-193b-5p is indicated with an arrow. [file 13020_2023_842_MOESM1_ESM.docx]

**
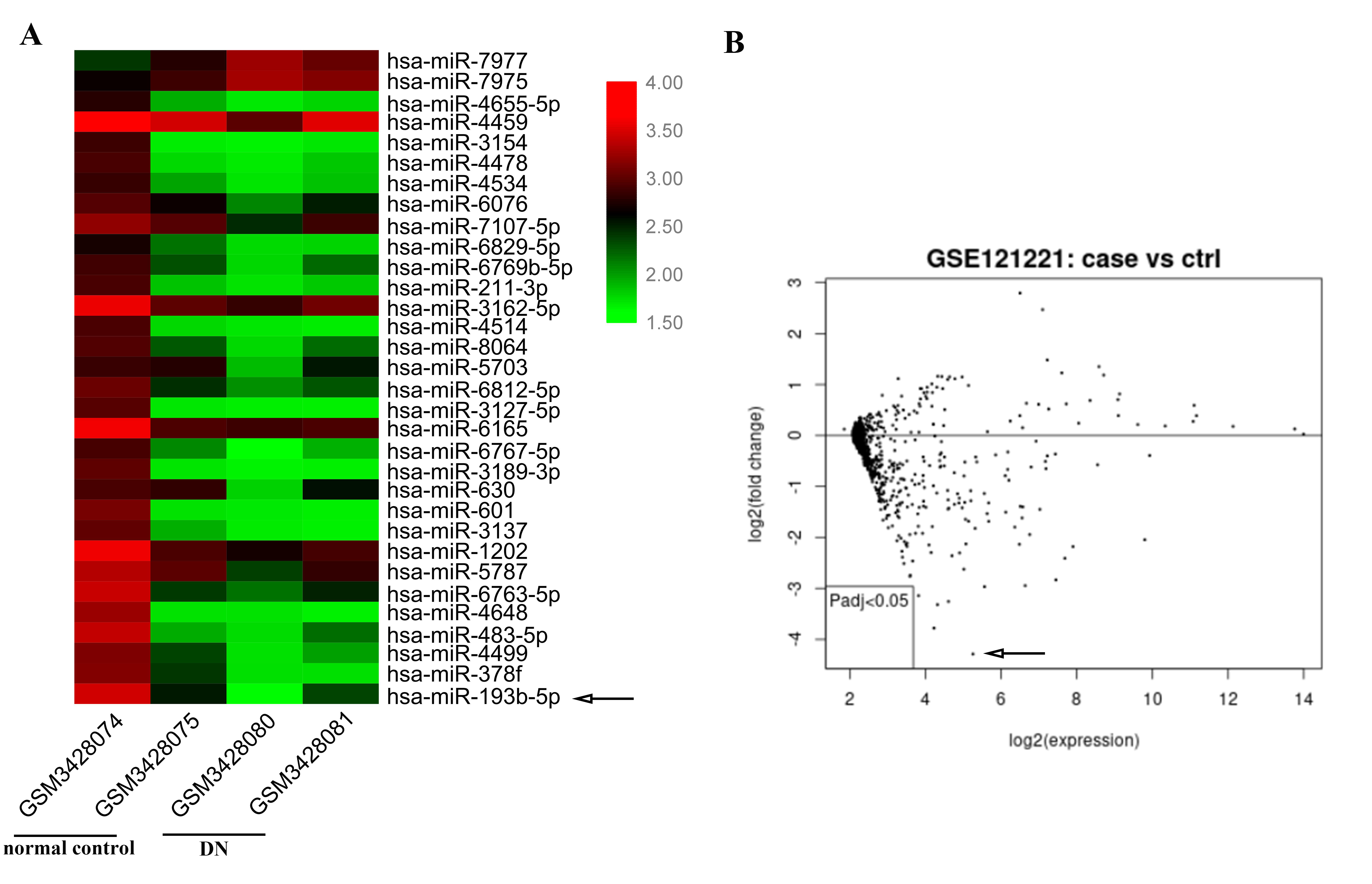
**

**Figure S1.** **MiR-193b-5p is down-regulated in DN patient.** **(A)** The cluster heatmap from microarray data (*P* < 0.05) (GEO121221) shows miRNAs in the mid-morning urine samples with different expression patterns among normal control and DN patients. Each column represents a sample, and each row represents a miRNA. miRNAs with a fold change ≥ 2 and *P* value ≤ 0.05 are shown in the heat map above. miR-193b-5p is marked with an arrow. **(B)** Volcano plot shows the up-regulated and down-regulated miRNAs in normal control and DN patient. miR-193b-5p is indicated with an arrow.
