# Supplementary material for: Cordycepin from Cordyceps militaris ameliorates diabetic nephropathy via the miR-193b-5p/MCL-1 axis
Source: Chin Med. 2023 Oct 13;18:134. doi: 10.1186/s13020-023-00842-5 (PMC10576278; doi:10.1186/s13020-023-00842-5)

Figure 1F

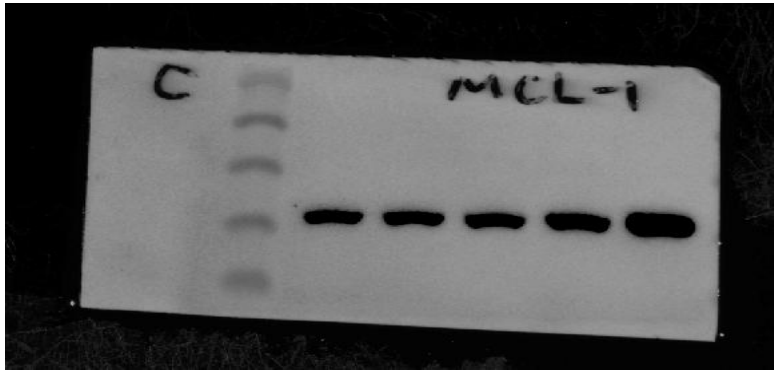

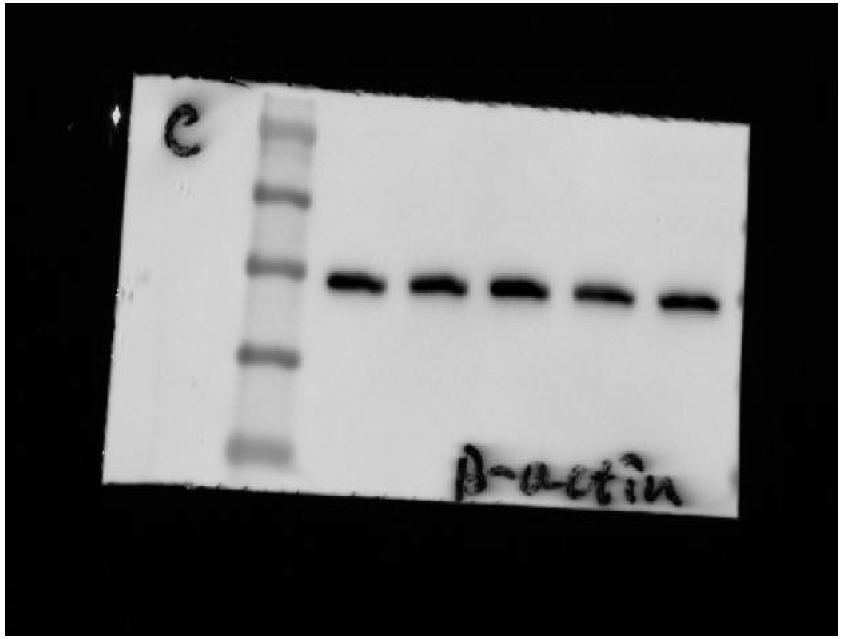

Figure 2E

PCNA

Cycilin D1

ACTIN

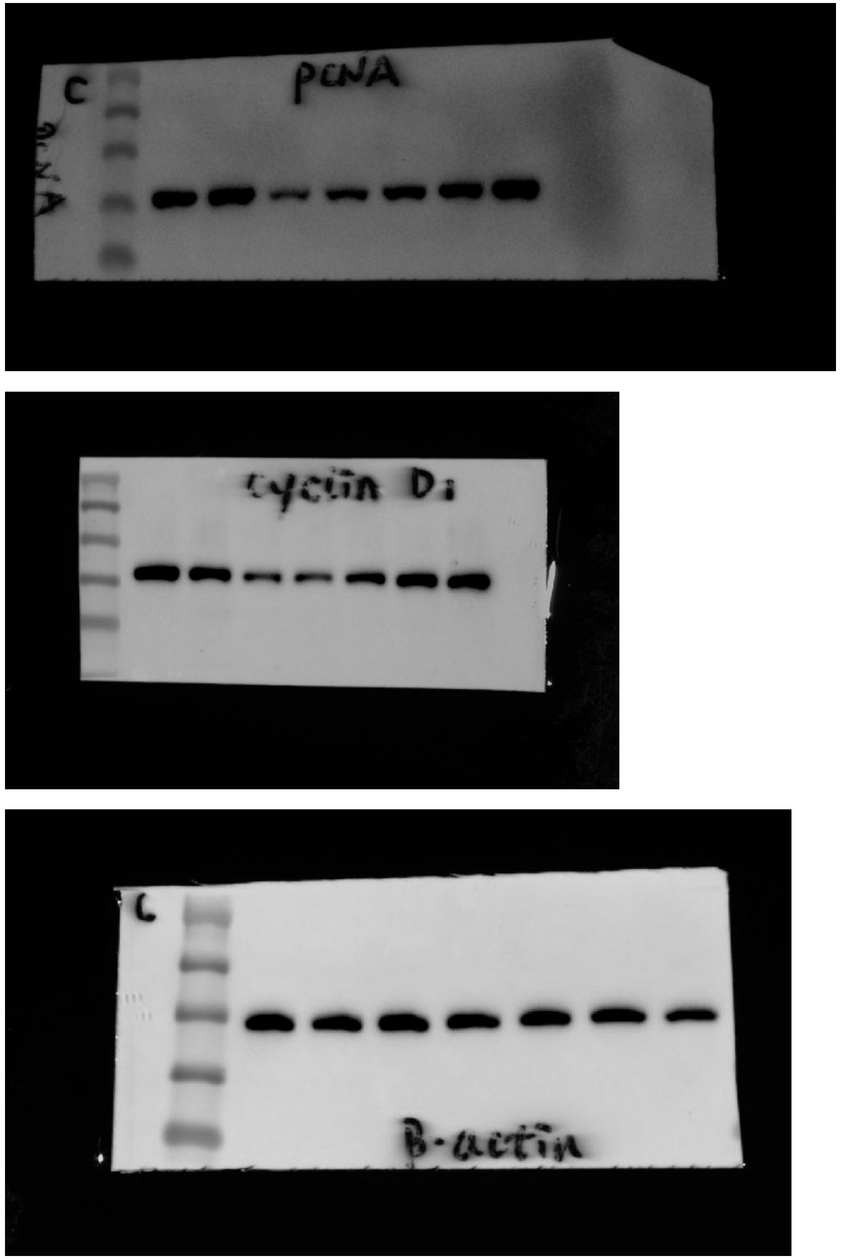

Figure 2J

BCL-2

BAX

cl-caspase-3

ACTIN

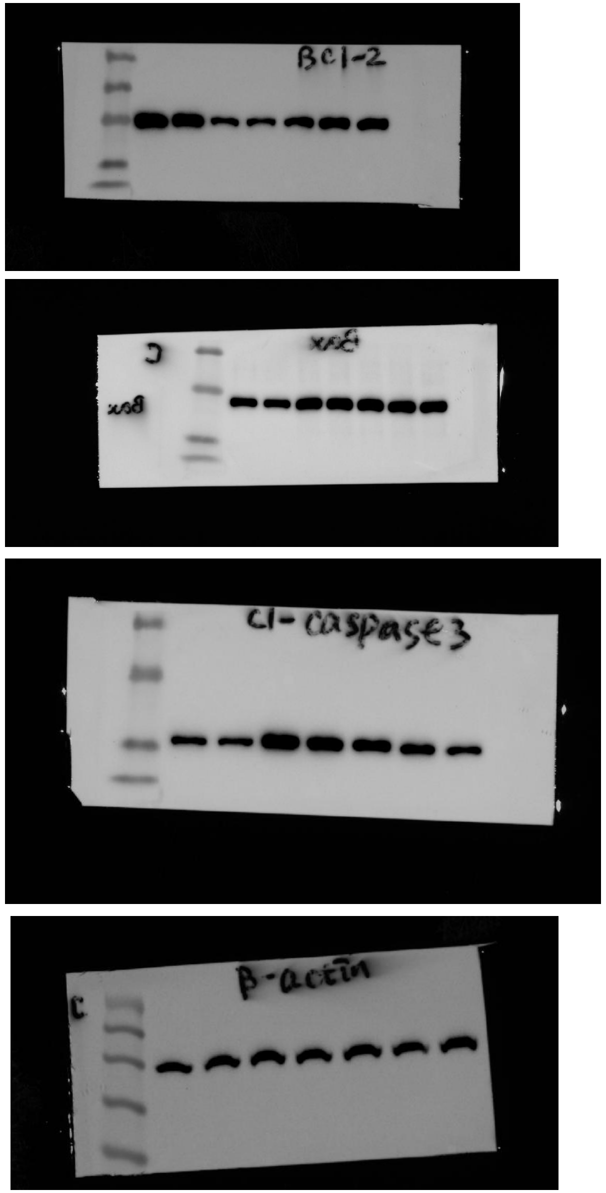

Figure 3D

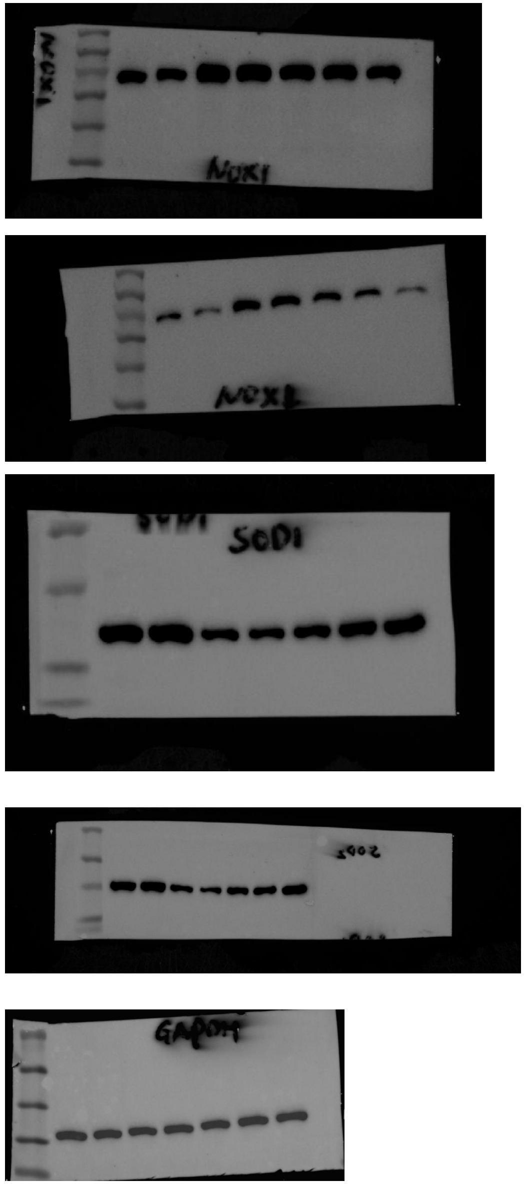

NOX1

NOX2

SOD1

SOD2

GAPDH

Figure 5F

MCL-1

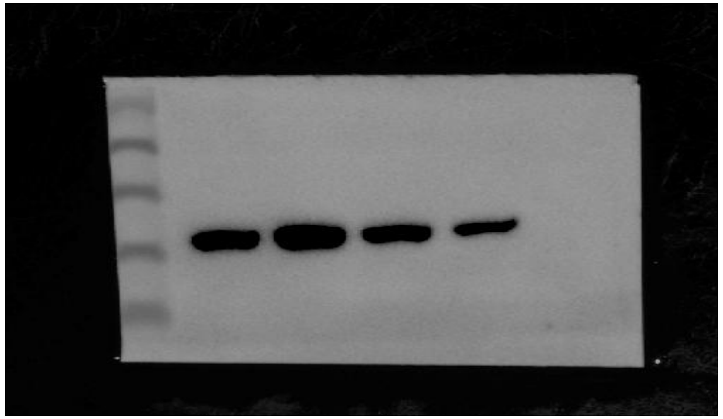

ACTIN

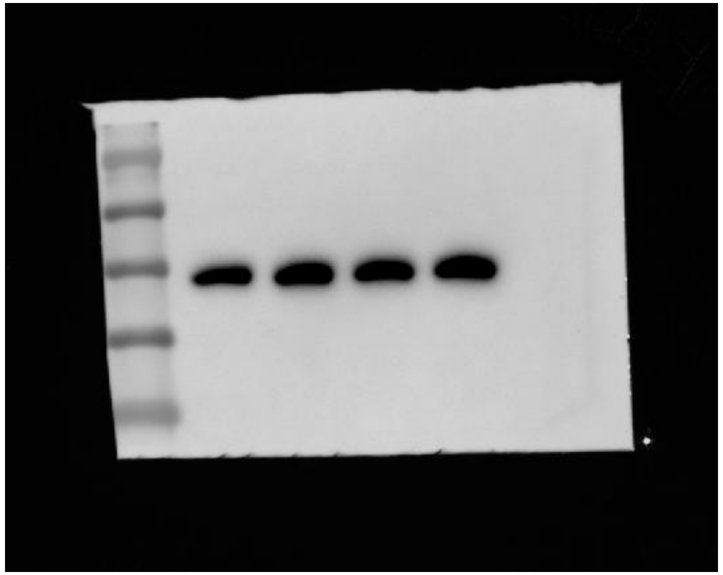

Figure 6B

MCL-1

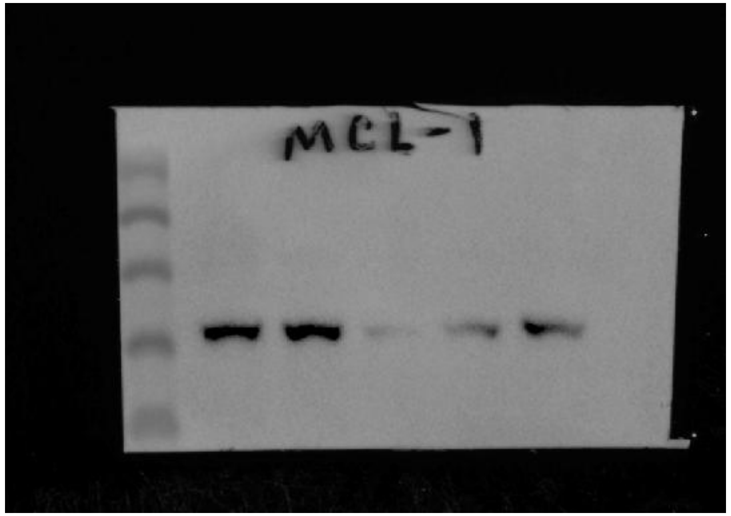

ACTIN

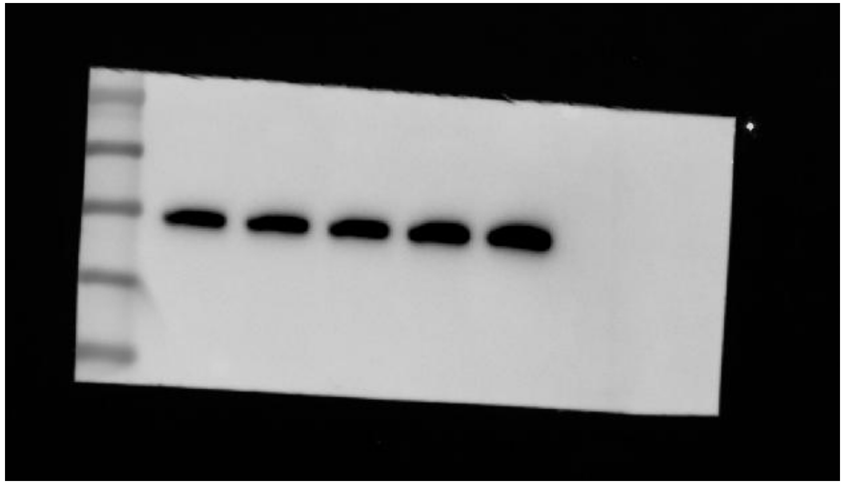

Figure 7L

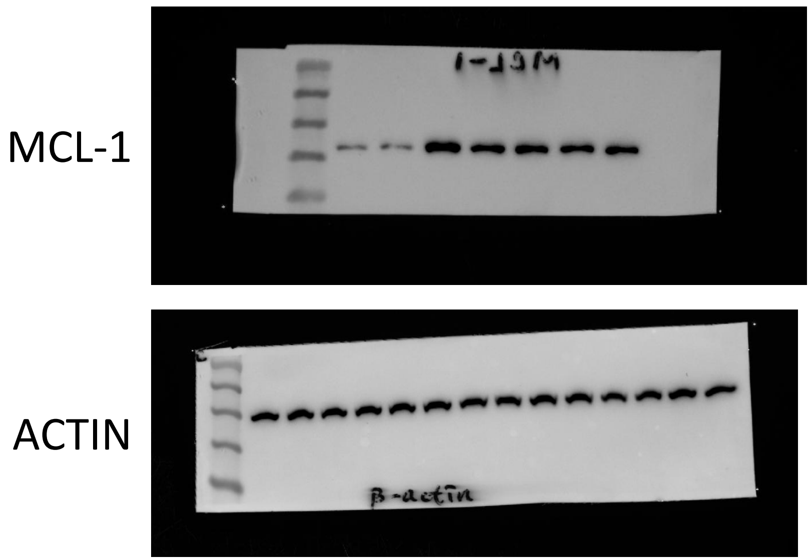

Figure 8M

Cl-CASPASE-3

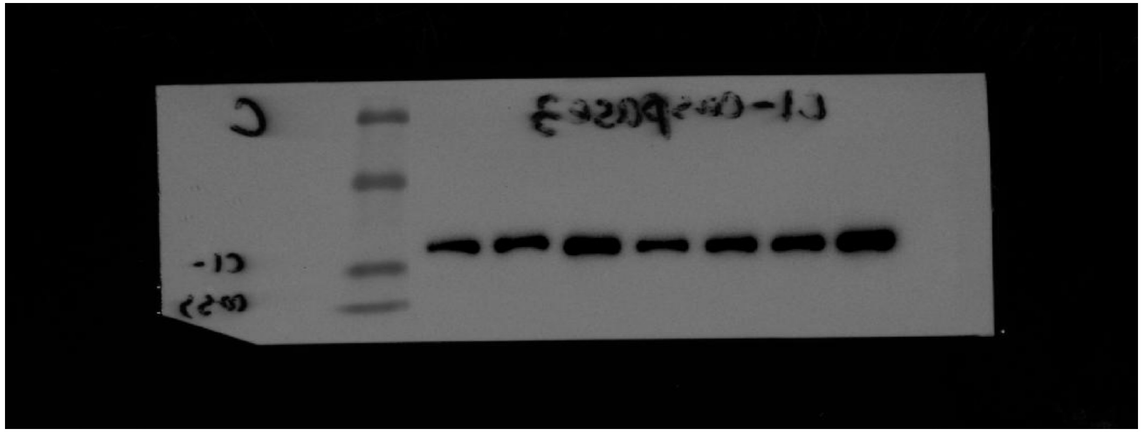

caspase-3

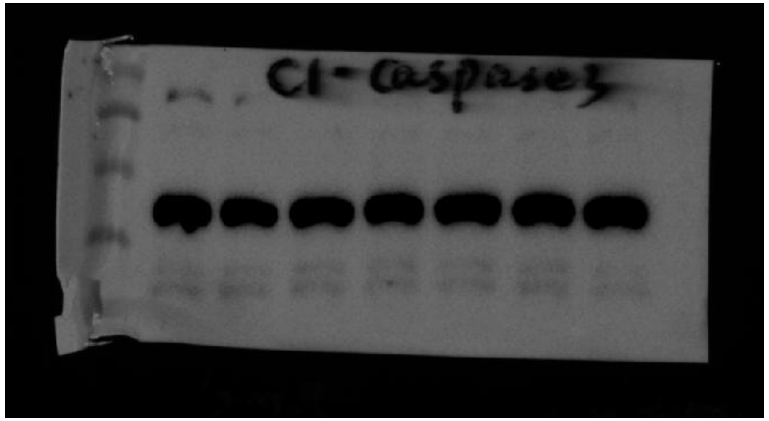

Supplement: Supplementary file 2 — Additional file 2. Compiled set of unedited images of the original membrane used in the WB experiments. [file 13020_2023_842_MOESM2_ESM.pdf]
